# iNOS modulates inflammatory responses in an NO-independent manner through direct interaction with IRG1 in mitochondria

**DOI:** 10.1038/s42255-026-01492-1

**Published:** 2026-04-10

**Authors:** Marina Diotallevi, Carlos Outeiral, Priyanka Patel, Gareth S. D. Purvis, Surawee Chuaiphichai, Thomas Nicol, Faseeha Ayaz, Daniel A. Nissley, Ganna O. Krasnoselska, Svenja Hester, John H. McVey, Roman Fischer, Benedikt Kessler, Charlotte M. Deane, Raymond J. Owens, Keith M. Channon, Mark J. Crabtree

**Affiliations:** 1 BHF Centre of Research Excellence, Division of Cardiovascular Medicine, Radcliffe Department of Medicine, John Radcliffe Hospital, University of Oxford, Oxford, UK; 2 Centre for Human Genetics, University of Oxford, Oxford, UK; 3 Oxford Protein Informatics Group, University of Oxford, Oxford, UK; 4 Department of Biochemical Sciences, School of Biosciences and Medicine, University of Surrey, Guildford, UK; 5 Structural Biology, Rosalind Franklin Institute, Harwell Science Campus, Didcot, UK; 6 Protein Production UK, The Research Complex at Harwell, Science Campus, Didcot, UK; 7 Target Discovery Institute, Nuffield Department of Medicine, University of Oxford, Oxford, UK; 8 Division of Structural Biology, Centre for Human Genetics, University of Oxford, Oxford, UK

**Keywords:** Mitochondrial proteins, Inflammation, Mitochondria

## Abstract

Nitric oxide (NO) has fundamental roles in numerous physiological and pathophysiological processes. In macrophages, NO produced by inducible nitric oxide synthase (iNOS) modulates metabolic changes that are essential to macrophage activation and plasticity, driving the characteristic metabolic switch from oxidative phosphorylation to glycolysis^1,2^. Itaconate, derived from the TCA cycle by decarboxylation of *cis*-aconitate by IRG1 (also referred to as CAD, ACOD1), is one of the most upregulated metabolites during the inflammatory response^3^. Itaconate regulates macrophage polarization by electrophilically modifying cysteines of key enzymes that control inflammatory states (such as ATF3, Jak1, IFNβ), participate in glycolysis (for example, GAPDH, LDHA) and limit oxidative stress through structural competitive inhibition of succinate dehydrogenase^4–9^. We recently reported that macrophages that are deficient in iNOS, and subsequent NO generation, produce strikingly higher levels of intracellular itaconate (up to ~15-fold) compared to wild-type cells when stimulated with inflammatory cytokines^1,2,10^. Here we show that iNOS inhibits IRG1 activity and itaconate levels through a conformation-dependent protein–protein interaction rather than through the production of NO. Using a variety of biochemical and computational approaches, we show that a direct interaction between iNOS and IRG1 occurs within mitochondria, in mouse and human cells, and that it depends on binding of the cofactor BH4 to iNOS but does not require its capability to produce NO. Our findings reveal a non-canonical cellular function for iNOS that places it at the centre of a signalling hub, linking redox signalling and metabolism to modulation of the inflammatory response in macrophages.

## Abstract (web summary)

This study reveals a non-canonical immunomodulatory function for the enzyme iNOS, based on the direct interaction in mitochondria with the itaconate-producing protein IRG1, which limits itaconate production in mouse and human macrophages

## Main

**Fig. 1:**
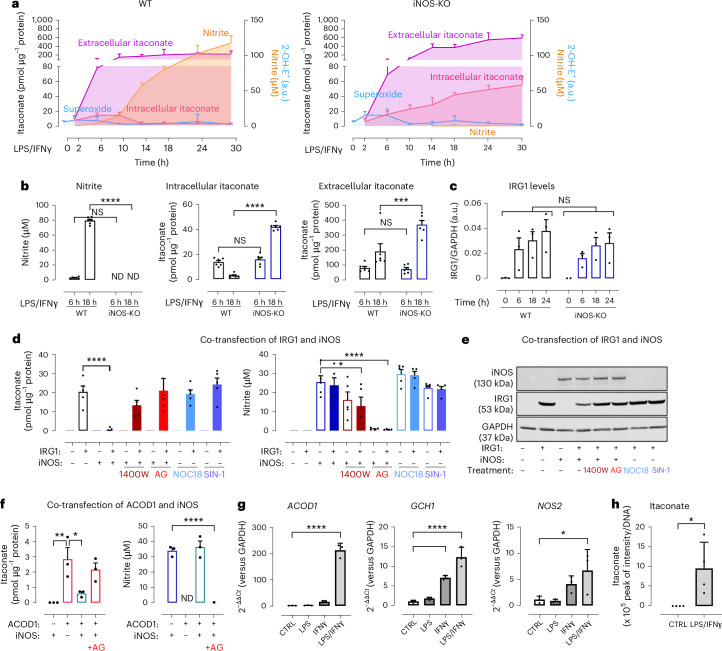
Itaconate accumulation is inhibited by iNOS. **a**, Itaconate levels measured in cell lysate (pink) or media (purple) from BMDM culture using HPLC at specific time points following LPS/IFNγ stimulation (*n* = 6 mice per group). Nitrite (orange) measured in media by Griess assay, and superoxide anion (blue) in cell pellets by HPLC from WT (*n* = 7 mice) and iNOS-KO (*n* = 4 mice) BMDMs. Data are presented as mean values; error bars, s.d. 2-OH-E^+^, 2-hydroxyethidium. **b**, Data at 6 h and 18 h were extracted and plotted as a bar chart. Statistical differences were calculated using a two-way analysis of variance (ANOVA) with Šídák’s multiple comparisons test. ND, not detected. **c**, Densitometry of IRG1/GAPDH is shown (*n* = 3 mice). Data are expressed as mean values; error bars, s.e.m. Statistical differences were determined by a two-way ANOVA with Šídák’s multiple comparisons test for each time point; see Extended Data Fig. 1c for a representative western blot. **d**, HEK cells transfected with iNOS and IRG1 cDNAs and treated with iNOS inhibitors (1400W (10 µM) or aminoguanidine (AG; 1 mM)) or NO donors (NOC18 (50 µM) or SIN-1 (100 µM)). Itaconate was measured in cell pellets and nitrite production in media. Data are represented in bar charts as means of *n* = 5 independent experiments; error bars, s.e.m. Statistical differences were calculated using one-way ANOVA with Dunnett’s multiple comparisons test against the IRG1 condition for itaconate measurement and against the iNOS condition for nitrite measurement. **e**, Representative western blot of *n* = 3 independent experiments showing protein levels of IRG1 and iNOS compared to the loading control GAPDH following co-transfection of HEK cells with IRG1 and iNOS cDNAs. **f**, Intracellular itaconate and nitrite measured in HEK cells transfected with human IRG1 (ACOD1) cDNA. Data are expressed as the mean of *n* = 3 independent experiments; error bars, s.e.m. Statistical differences were calculated using a one-way ANOVA with Dunnett’s multiple comparisons test. **g**, Relative gene expression of *ACOD1*, *NOS2* and *GCH1* in human bone marrow organoids compared with *GAPDH* control. Data are represented in bar charts as the means of *n* = 3 biological replicates; error bars, s.d. Statistical differences were calculated using a one-way ANOVA with Dunnett’s multiple comparisons test against the CTRL condition. **h**, Intracellular levels of itaconate in human bone marrow organoids measured by mass spectrometry. Data are represented in bar charts as the mean values of *n* = 3 biological replicates; error bars, s.d. Statistical differences were calculated using an unpaired *t*-test. Statistical significance is indicated as ****P < 0.0001; ***P < 0.001; **P < 0.005; *P < 0.05; NS, not significant. Source data

**Extended Data Fig. 1:**
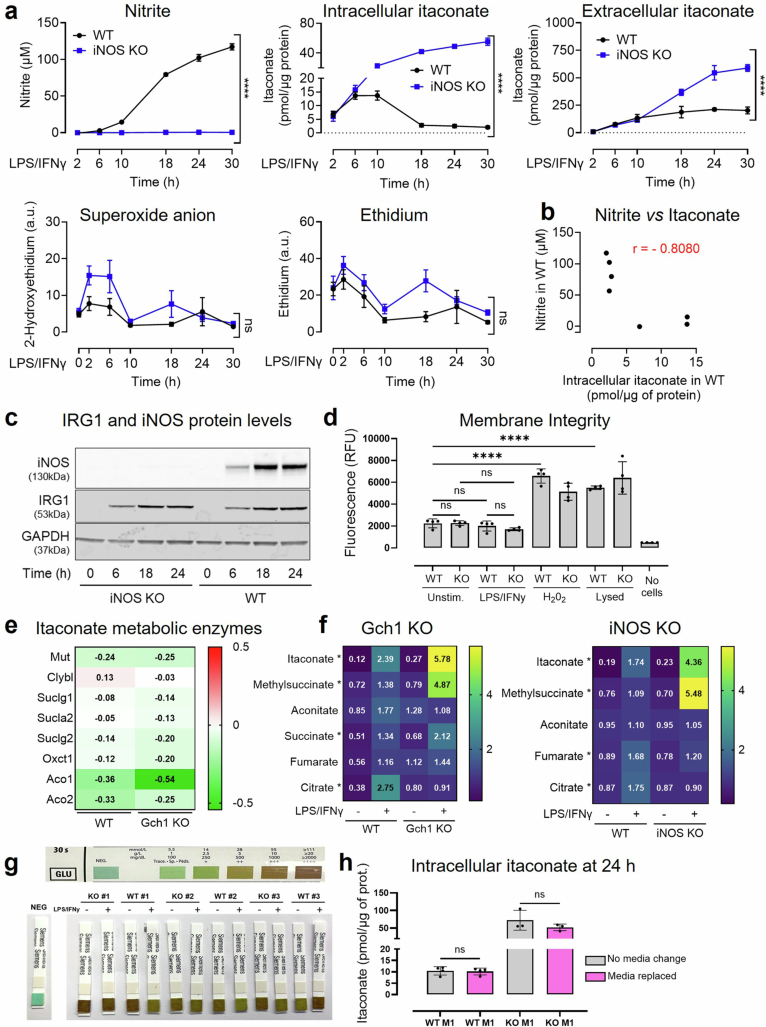
Itaconate increase in iNOS KO is not due to alteration of itaconate metabolism. **a**, Itaconate levels were measured in the cell lysate or media from BMDM culture using HPLC at specific time points following LPS/IFNγ stimulation (n = 6 mice per group). Nitrite was measured in media by Griess assay, and superoxide anion in cell pellets by HPLC from WT (n = 7 mice) and iNOS KO (n = 4 mice) BMDMs. All data is expressed as mean values ± SEM. Statistical differences were calculated as a two-way ANOVA with multiple comparisons test and indicate the variation between genotypes over time. **b**, Pearson correlation (r) comparing nitrite production from WT vs. intracellular itaconate at each time point (black dot). **c**, Representative Western blot of n = 3 mice per group; BMDMs following 6, 18 and 24 h exposure to LPS/IFNγ, probed with anti-iNOS, anti-IRG1 and anti-GAPDH (loading control). **d**, Membrane integrity was assessed using CellTox™ Green Cytotoxicity Assay (Promega). Cells were seeded at 50,000 cells in a 96-well plate and cultured for 24 h with LPS/IFNy or 100 µM H202 or lysed 30 min prior the assay as indicated. Data are presented as mean values of n = 4 mice per group ± SD. Statistical differences were calculated as one-way ANOVA with Dunnett’s multiple comparisons. **e**, Heatmap of fold change of protein abundance linked with itaconate catabolism / anabolism of LPS/IFNγ from Bailey et al. (2019). Data are expressed as fold-change of M(^LPS/IFNγ^) versus unstimulated macrophages M(^unstimulated^) and a scale indicating the fold change is shown (n = 4 mice per group); two-way ANOVA followed by Tukey HSD method to create a set of confidence intervals based on the sample means. Proteins having a P-value < 9×10^−6^ (based on Bonferroni correction = 0.05/5704) were considered significant. **f**, Heatmap of normalised abundance values for metabolites for both genotypes from Bailey et al. (2019); Gch1, iNOS KO and WT following 18 h stimulation with LPS/IFNγ. Scale indicate metabolites abundance (n = 6 mice per group), a two-way ANOVA was performed between genotype group. **g**, Glucose levels were assessed in cell media at 24 h from samples used in Fig. 1a,b using glucose strips ranging from 0 g/L to >20 g/L. **h**, Intracellular itaconate measured by HPLC 24 h following media change at 22 h (pink bar) or not (grey bar). Data are presented as mean values of n = 3 or 4 mice per group ± SD. Statistical differences were calculated as one-way ANOVA with Tukey’s multiple comparisons. Statistical significance is indicated as ****p < 0.0001, ***p < 0.001, **p < 0.005, *p < 0.05, ns= non-significant. Source data

To understand the role of inducible nitric oxide synthase (iNOS) and nitric oxide (NO) in modulating itaconate production during inflammation, we compared the nitroso-redox profile of bone marrow-derived macrophages (BMDMs) from wild-type (WT) mice with those from iNOS knockout (iNOS-KO) mice at various time points (0–30 h) after LPS/IFNγ treatment^1^ (Fig. 1a–c and Extended Data Fig. 1a–c). As expected, LPS/IFNγ stimulation induced abundant NO production, demonstrated by nitrite accumulation, in media from WT cells but not in media from iNOS-KO cells. Superoxide production was increased in iNOS-KO cells versus WT cells after stimulation, reflecting loss of iNOS-derived NO production^10^. Similar levels of intracellular and extracellular itaconate were produced by both iNOS-KO cells and WT cells up to 6 h, but in the later stages of activation (6–30 h), striking differences in iNOS-dependent redox changes were observed. Intracellular itaconate levels decreased in WT cells, consistent with other reports^11^, whereas iNOS-KO cells continued to accumulate intracellular itaconate, reaching more than a 15-fold difference after 18 h. The difference in extracellular itaconate was even greater. Extracellular itaconate from WT cells plateaued at approximately 200 pmol µg^−1^ of protein while no ‘brake’ occurred in iNOS-KO macrophages, with levels of extracellular itaconate rising to ~370 pmol µg^−1^ protein at 18 h (Fig. 1b). In the WT cells, reduction of intracellular itaconate correlated with accumulation of nitrite over time following LPS/IFNγ treatment, as supported by a strong negative Pearson correlation coefficient (*r* = –0.81, *P* = 0.028), suggesting a tight association between NO generation and itaconate production (Extended Data Fig. 1b). As no change in either IRG1 protein abundance or cell viability was observed between genotypes (Fig. 1c and Extended Data Fig. 1c,d), we reasoned that iNOS and/or NO control itaconate production by affecting IRG1 activity or by targeting itaconate metabolism^9,12^. Proteomic and metabolic analyses of both iNOS-deficient BMDMs or cells deficient in the iNOS cofactor tetrahydrobiopterin (BH4) (also referred to as *Gch1*-KO BMDMs)^1^ demonstrated that itaconate catabolic enzymes such as CLYBL, MUT and OXCT1 remained unaltered, while downstream metabolites of itaconate in NO-deficient macrophages (for example, methylsuccinate, succinate) were increased rather than diminished in NO-deficient macrophages, indicative of maintained itaconate catabolism (Extended Data Fig. 1e,f). Levels of aconitate, the substrate for itaconate production by IRG1, remained unchanged in iNOS-KO BMDMs compared to WT BMDMs, hinting that aconitate bioavailability could not explain the accumulation of itaconate. Similarly, glucose levels in cell media at 24 h remained >2.5 g l^−1^ in all samples, implying that glucose depletion could not account for the lower levels of itaconate observed in WT versus iNOS macrophages (Extended Data Fig. 1g). Furthermore, replacing media at 22 h to replenish cells with fresh nutrients did not alter itaconate levels at 24 h (Extended Data Fig. 1h). Taken together, these results suggest that the difference in itaconate production in iNOS-KO macrophages is not caused by limiting factors in the culture media.

To test the mutual requirements for itaconate production by IRG1 in the presence or absence of iNOS, we transfected human embryonic kidney (HEK) cells with iNOS and IRG1 cDNA (Fig. 1d,e), as HEK cells are known to be devoid of both endogenous iNOS and IRG1. Overexpression of IRG1 in HEK cells led to intracellular itaconate production at similar levels to those observed in stimulated WT macrophages (~20 pmol µg^−1^ protein at 6 h). Transfection with iNOS led to substantial NO synthase activity, as evidenced by marked nitrite accumulation. Co-transfection with both iNOS and IRG1 abolished the production of itaconate completely, while NO production remained unaffected. These observations in transfected HEK cells reproduce the inhibitory effect of iNOS on itaconate synthesis observed in WT primary macrophages. Similarly, IRG1 protein levels remained unchanged in the presence or absence of iNOS, confirming that iNOS affects IRG1 catalytic activity (Fig. 1e).

Given that iNOS is sufficient to inhibit itaconate production, we explored whether inhibiting iNOS activity would restore itaconate levels, using two different inhibitors of iNOS: 1400W dihydrochloride (1400W) and aminoguanidine (AG). Although AG led to complete depletion of nitrite and full restoration of itaconate production, 1400W only reduced nitrite production by one-third, but significantly rescued itaconate production (Fig. 1d). NOC18 and SIN-1, donors of NO and peroxynitrite, respectively, had no inhibitory effect on itaconate levels (Fig. 1d). These observations demonstrate a discordance between NO production and IRG1 inhibition, suggesting a further mechanism in addition to biochemical NO effects alone.

**Extended Data Fig. 2:**
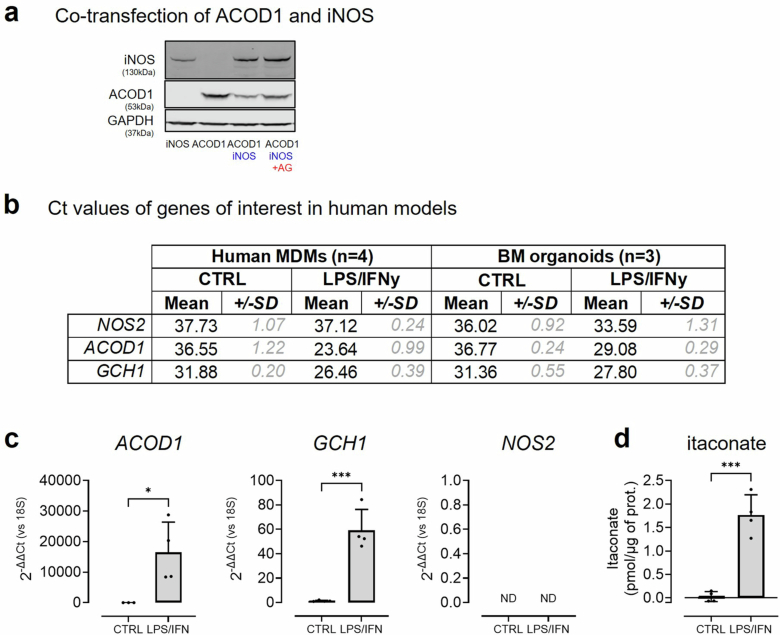
iNOS and IRG1 in human macrophages. **a**, Representative Western blot of 3 showing proteins levels of IRG1 and iNOS measured in HEK cells transfected with human IRG1 (hIRG1) cDNA. **b**, Table of cycle threshold (Ct) values for each gene of interest (*NOS2, ACOD1*, and *GCH1*) in unstimulated (CTRL) or 24 h LPS/IFNγ stimulated of n = 4 human monocyte-derived macrophages (Human MDMs) or n = 3 bone marrow organoids (BM organoids). **c**, Relative gene expression of *ACOD1*, *NOS*2 and *GCH1* in human MDMs compared to control. Data is represented in bar charts as mean of n = 4 biological replicates ± SD. Statistical differences were calculated as an unpaired t-test. **d**, Intracellular levels of itaconate in human MDMs measured by HPLC. Data is represented in bar charts as mean of n = 4 biological replicates ± SD. Statistical differences were calculated as an unpaired t-test. Statistical significance is indicated as ****p < 0.0001, ***p < 0.001, **p < 0.005, *p < 0.05, ns= non-significant. Source data

HEK cells were also transfected with human IRG1 (ACOD1) and co-transfected with iNOS (Fig. 1f and Extended Data Fig. 2a). Although ACOD1 produced lower levels of itaconate than mouse IRG1, similar inhibition by iNOS and rescue with AG were observed. We next sought to determine whether a similar mechanism could be observed in human macrophages. We tested human monocyte-derived macrophages (hMDMs) derived from peripheral blood mononuclear cells (PBMCs) in the presence of GM-CSF, a stimulating factor known to increase NO in murine cells^13^. Despite the induction of *GCH1* (which encodes GTP cyclohydrolase 1, leading to the synthesis of BH4) and *ACOD1* expression, as well as the detection of itaconate following LPS/IFNγ stimulation, *NOS2* expression (encoding iNOS) was not induced, and nitrite levels were not increased in human macrophages (Extended Data Fig. 2b–d). These results support previous studies showing that *NOS2* is epigenetically silenced in cultured human macrophages, despite using a variety of pro-inflammatory stimuli^14^. Although many studies have reported elevated iNOS mRNA and protein levels in tissues from patients with various chronic diseases, detection of iNOS in human cells in vitro has been inconsistent^15–20^. To overcome this constraint, we used a previously developed human inducible pluripotent stem cell (iPS cell)-derived bone marrow organoid system^21^, offering greater biological complexity enhanced by cell–cell interaction. This system produces mature monocytic cells, derived from hematopoietic stem cells in a niche supported by endothelial and stromal cells. We demonstrated that *NOS2* is induced upon IFNγ and LPS/IFNγ stimuli for 24 h alongside *ACOD1* and *GCH1* (Fig. 1g,h). Intracellular itaconate was detected upon LPS/IFNγ stimulation (Fig. 1h), providing evidence that *NOS2* and *ACOD1* are both expressed under similar pro-inflammatory settings in human myeloid cells.

**Fig. 2:**
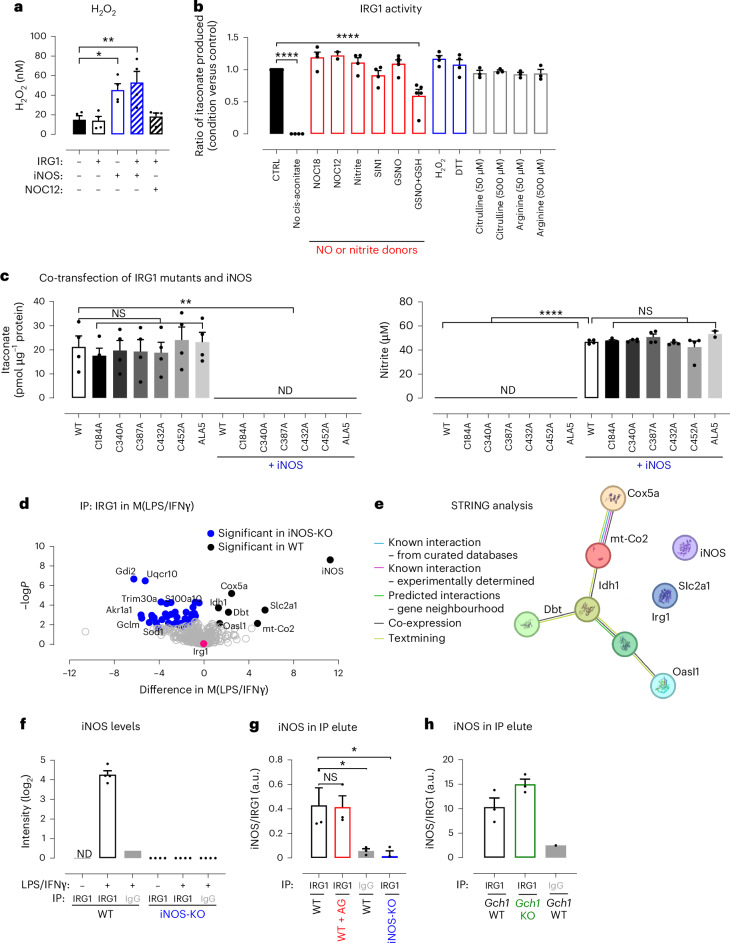
IRG1 is regulated by iNOS but not by NO. **a**, Hydrogen peroxide levels were determined in media following HEK cell transfection with IRG1 or iNOS cDNA, in the presence or absence of NOC12. Data are expressed as mean values of *n* = 4 technical replicates; error bars, s.e.m. Statistical differences were calculated using a one-way ANOVA with Dunnett’s multiple comparisons test. **b**, Activity of purified mouse IRG1 (detailed in Methods) measured by HPLC following incubation with different NO and nitrite donors: 1 mM nitrite, 1 mM SIN-1, 1 mM NOC12 and NOC18, 1 mM GSNO (+10 mM of GSH or GSSG), 1 mM H_2_O_2_, 1 mM DTT, 50 or 500 µM of citrulline and 50 or 500 µM of arginine. Data are expressed as mean values of *n* = 3–4 independent experiments; error bars, s.e.m. Statistical differences were calculated using a one-way ANOVA with Dunnett’s multiple comparisons test against the CTRL condition. **c**, HEK cells were transfected with IRG1 WT or its mutants cDNA (C184A, C340A, C387A, C432A, C452A, ALA5) and both intracellular itaconate and nitrite levels were measured in the presence or absence of iNOS cDNA. Data are expressed as mean values of *n* = 4 independent experiments; error bars, s.e.m. Statistical differences were calculated using a one-way ANOVA with Dunnett’s multiple comparisons test against WT or WT + iNOS, respectively. **d**, Volcano plot of proteins pulled down by Co-IP and identified by mass spectrometry in M(LPS/IFNγ) showing proteins significantly associated with IRG1 in WT (black dots) or iNOS-KO (blue dots) (*n* = 4 mice per group) following a two-tailed Student’s *t*-test set at 0.5. **e**, IRG1 interactome from WT BMDMs was exposed to STRING analysis to exhibit known or predicted interactions using medium-confidence (0.400) settings. **f**, iNOS intensity data from mass spectrometry were extracted and expressed as a bar chart of mean values of *n* = 4 independent experiments; error bars, s.e.m. **g**, Densitometry of iNOS/IRG1 in IP elute in the iNOS-KO model (*n* = 3 mice per group). IRG1 was precipitated from the cell pellet. Western blots (Extended Data Fig. 4g) were pre-incubated with red fluorophore secondary anti-IgG rabbit and then re-incubated with rabbit anti-IRG1 (using a green secondary antibody), allowing visual separation of IRG1 (53 kDa) from IgG heavy chains present in the immunoprecipitate. Data are expressed as the mean of *n* = 3 mice; error bars, s.e.m. Statistical differences were calculated using a one-way ANOVA with Dunnett’s multiple comparisons test against WT. **h**, Densitometry of iNOS/IRG1 in IP elute in the *Gch1*-KO model (*n* = 3 mice). IRG1 was precipitated from the cell pellet. Western blots (Extended Data Fig. 4h) were pre-incubated with red fluorophore secondary anti-IgG rabbit and then re-incubated with rabbit anti-IRG1 (using a green secondary antibody), allowing visual separation of IRG1 (53 kDa) from IgG heavy chains in the immunoprecipitate. Data are expressed as the mean of *n* = 3 mice; error bars, s.e.m. Statistical differences were calculated using Dunnett’s multiple comparisons test against WT. Statistical significance is indicated as ****P < 0.0001; **P < 0.005; *P < 0.05. Source data

**Extended Data Fig. 3:**
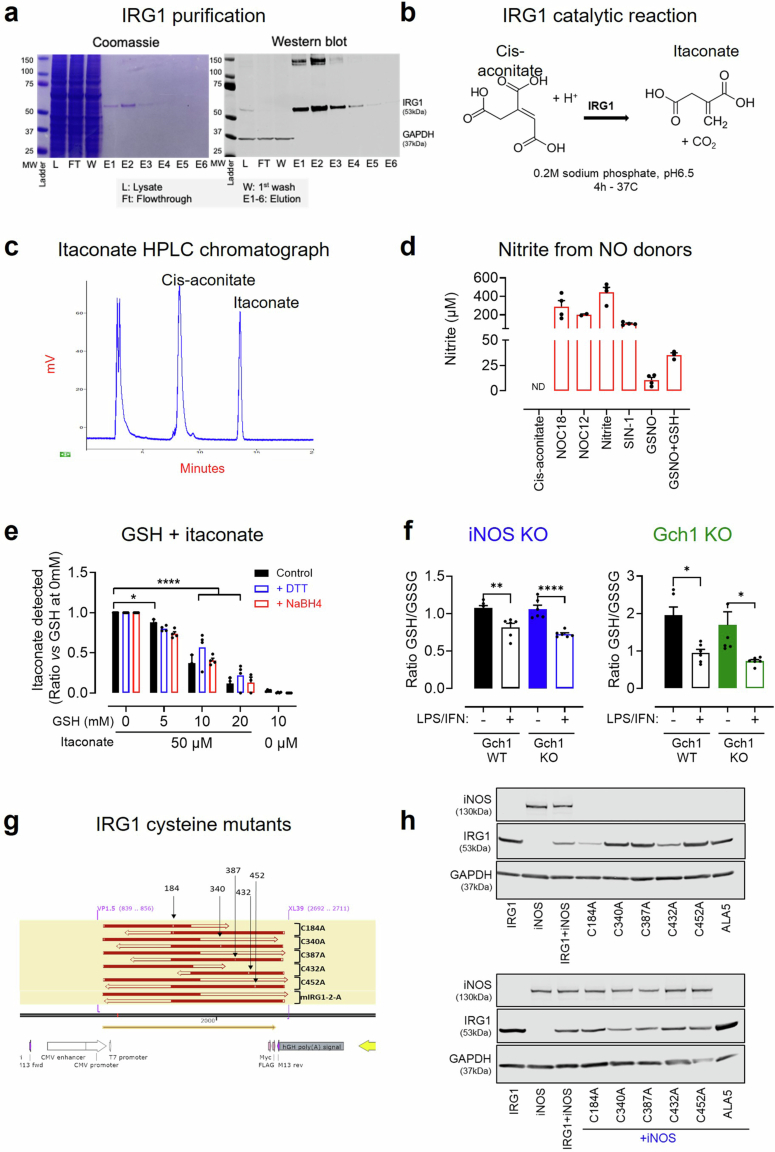
IRG1 is not regulated by NO. Mouse IRG1 was purified as detailed in Material & Methods. **a**, IRG1 purification quality is assessed by Coomassie and Western blotting. **b**, The activity of purified mouse IRG1 was determined by UV, using HPLC at 210 nm and measured 4 h following addition of cis-aconitate at 37 °C in a 0.2 M sodium phosphate solution. **c**, HPLC chromatograph example of cis-aconitate and itaconate standard detected by HPLC at 210 nm. **d**, Corresponding nitrite levels of Fig. 2b measured for each condition involving NO production; data is expressed as mean of n = 4 independent experiments ± SEM. **e**, Effect of increasing concentration of GSH on itaconate standard set at 50 mM. Data are expressed as mean of n = 4 independent experiments ± SEM; Statistical differences were calculated as a one-way ANOVA with Dunnett’s multiple comparisons test against Itaconate standard with no GSH. **f**, GSH/GSSH ratio were extracted from metabolomic analysis performed in Gch1 WT and Gch1 KO BMDMs and iNOS KO and WT following 18 h stimulation with LPS/IFNγ^1^. Data is plotted as mean values of n = 6 mice per group ± SEM and show the ratio of GSH/GSSG in both M(^LPS/IFNγ^) versus unstimulated macrophages (M(^unstimulated^)) for both genotypes. A one-way ANOVA with Tukey’s mutliple comparisons test was performed. **g**, Each cysteine of mCAD was mutated individually and replaced with alanine. A mutant was also created with all cysteines mutated to alanines (ALA5). Mismatches are shown by a small white bar and indicated with arrows. **h**, Protein levels of IRG1 WT and mutants, iNOS and GAPDH (loading control) following transfection were assessed by Western blotting (n = 3 independent experiments). Statistical significance is indicated as ****p < 0.0001, ***p < 0.001, **p < 0.005, *p < 0.05, ns= non-significant. Source data

We next investigated the role of iNOS-derived products, such as NO or nitrite, in regulating IRG1 activity. In addition, we observed that hydrogen peroxide (H_2_O_2_) was increased in media from HEK cells transfected with iNOS by a striking threefold increase compared to non-transfected cells (Fig. 2a). Given that NO and H_2_O_2_ are important signalling molecules that can alter protein structure and function by targeting specific redox-sensitive amino acid residues such as cysteine^22,23^, we investigated whether iNOS-derived NO or H_2_O_2_ could act through post-translational modification to inhibit the activity of IRG1. A high-performance liquid chromatography (HPLC)-based in vitro assay was developed to quantify the activity of recombinant IRG1 protein purified from a mammalian expression system (Methods and Extended Data Fig. 3a–c). Although itaconate production was detected in the presence of IRG1 and its substrate *cis*-aconitate (Fig. 2b), neither spontaneous NO donors such as NOC18 or NOC12, nor sodium nitrite, *S*-nitrosoglutathione (GSNO), SIN-1, H_2_O_2_, dithiothreitol (DTT), citrulline or arginine were able to attenuate itaconate production by IRG1, validating our findings in Fig. 1e. GSNO was also tested in the presence of reduced glutathione (GSH), as GSH is known to enhance NO production^24^ (Extended Data Fig. 3d). Although a significant reduction in itaconate levels in the presence of GSNO + GSH was observed, recent studies have reported that GSH directly reacts with itaconate forming an itaconate–GSH adduct^25^. This was further demonstrated by measuring a significant decrease in standard itaconate when exposed to increasing concentrations of GSH, suggesting the formation of an itaconate–GSH adduct, with distinct chemical properties not detected by the HPLC assay (Extended Data Fig. 3e). As the GSH/GSSG ratio was also unaltered in stimulated macrophages from *Gch1*-KO and iNOS-KO mice, we predicted that the regulation of itaconate by glutathione could not explain the iNOS-dependent inhibition of IRG1 (Extended Data Fig. 3f). To confirm that iNOS-dependent NO or related reactive species such as glutathione do not inhibit IRG1 through post-translational modification of its redox-sensitive cysteines, we generated six IRG1 cysteine mutants (C184A, C340A, C387A, C432A, C452A and a combined mutant (ALA5)) (Fig. 2c and Extended Data Fig. 3g,h). All IRG1 cysteine mutants produced itaconate at WT IRG1 levels and remained fully inhibited by iNOS, indicating that inhibition of IRG1 occurs through cysteine-independent mechanisms.

**Extended Data Fig. 4:**
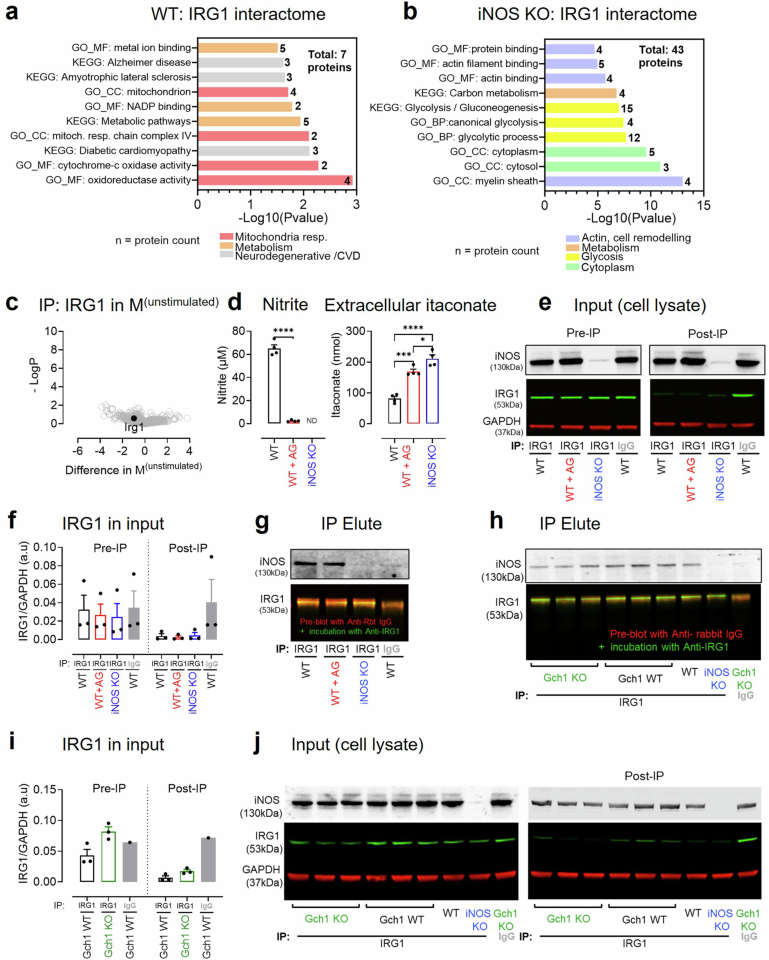
iNOS interacts with IRG1 independent of enzymatic activation. **a,b** Proteins identified in the WT (**a**) or iNOS KO IRG1 (**b**) -interactome were subjected to Gene Set Enrichment Analysis in DAVID using Gene Ontology (Biological Process (BP), Molecular Function (MF) and Cellular Component (CC)) and KEGG pathways databases. Categories obtained were filtered by ranking P-values and the ten first are shown here (See Extended Data Table 1 for full list of proteins). **c**, Volcano plot of M(^unstimultaed^) (Fold change (FC) / Log P value) from mass spectrometry data shows proteins significantly associated with IRG1 in WT or iNOS KO following a a two-tailed student T-test set at 0.5. **d**, Nitrite and extracellular itaconate levels in samples from iNOS KO, WT and WT in the presence of AG from BMDMs all stimulated with LPS/IFNγ. Data are presented as mean values of n = 4 mice per group ± SEM. Statistical differences were calculated as a one-way ANOVA with Dunnett’s multiple comparisons test. **e**, IRG1 was precipitated from cell pellet in the iNOS KO model. Input (Pre and post-IP lysate) and IP elute were subjected to Western blotting using rabbit anti-iRG1, mouse anti-GAPDH and mouse anti-iNOS**. f**, Densitometry of IRG1/GAPDH is shown as mean values of n = 3 mice ± SEM. **g**, For the IP elute, Western blot (n = 3 mice) was pre-incubated with red fluorophore secondary anti-IgG rabbit and then re-incubate with rabbit anti-IRG1 (using a green secondary antibody) allowing to separate visually IRG1 (53 kDa) to IgG heavy chains from IP. **h**, IRG1 was also precipitated out of cell pellets from Gch1 WT (n = 3 mice) and Gch1 KO BMDMs (n = 3 mice) stimulated with LPS/IFNγ for 18 h, and IP elute directly subjected to Western blotting as detailed previously. **i**, Densitometry of IRG1/GAPDH is shown as mean values of n = 3 mice ± SEM. **j**, Input (Pre and post-IP lysate) from Gch1 KO model (n = 3 mice per group) were subjected to Western blotting using rabbit anti-iRG1, mouse anti-GAPDH and mouse anti-iNOS. Statistical significance is indicated as ****p < 0.0001, ***p < 0.001, **p < 0.005, *p < 0.05, ns= non-significant. Source data

Having shown that iNOS activity is essential for the inhibition of IRG1 activity and modulation of intracellular itaconate levels, but without a direct dependence on NO effects, we next sought to investigate other mechanisms linking iNOS with the regulation of IRG1. We performed co-immunoprecipitation (Co-IP) experiments in WT and iNOS-deficient BMDMs stimulated with LPS/IFNγ, using mass spectrometry to identify protein binding partners of IRG1 (Fig. 2d–f, Extended Data Fig. 4a–c and Extended Data Table 1). In unstimulated macrophages, the IRG1 protein was not induced, so no binding partners were identified (Extended Data Fig. 4c). In stimulated macrophages, 51 proteins were significantly different between WT and iNOS-KO cells (Fig. 2d and Extended Data Table 1), while those identified in both iNOS-KO and WT cells were linked with cell remodelling, RNA translation and immune response. Among significantly different proteins, 43 specifically associated with IRG1 in the absence of iNOS (KO interactome) and were part of cytosolic and glycolysis-enriched pathways, as they include pyruvate kinase (*Pkm*), glycolytic enzymes and redox enzymes such as superoxide dismutase (*Sod1*) (Extended Data Fig. 4b and Extended Data Table 1). By contrast, only seven proteins were significantly present in the WT interactome (Fig. 2d,e and Extended Data Fig. 4a). Critically, one of these IRG1-binding proteins was iNOS (*Nos2*), which was by far the most abundant IRG1-binding partner (fold change of >11; Fig. 2f) and was not identified in the IgG control pull-down. Other IRG1-binding partners included inner mitochondrial proteins such as mitochondrially encoded cytochrome *c* oxidase II (*mt-Co2*), cytochrome *c* oxidase subunit 5a (*Cox5a*) and Dbt, important for the transfer of alpha-keto acid to coenzyme, as well as cytoplasmic isocitrate dehydrogenase 1, another TCA-derived enzyme. STRING analysis further confirmed known or predicted interactions between six of seven of the iNOS WT interactome (Acod1 (=IRG1), Dbt, Oasl1, Idh1, mt-Co2 and Cox5a), supporting and validating our Co-IP findings (Fig. 2e). To determine whether iNOS catalytic activity is required for interaction with IRG1, we further performed IRG1 pull-downs in WT BMDMs treated with the iNOS inhibitor AG (Fig. 2g and Extended Data Fig. 4d–g) and in *Gch1*-KO macrophages lacking NO production (Fig. 2f and Extended Data Fig. 4h–j). In both models, iNOS co-immunoprecipitated with IRG1 regardless of NO synthesis, indicating that iNOS catalytic activity is not required for interacting with IRG1.

**Fig. 3:**
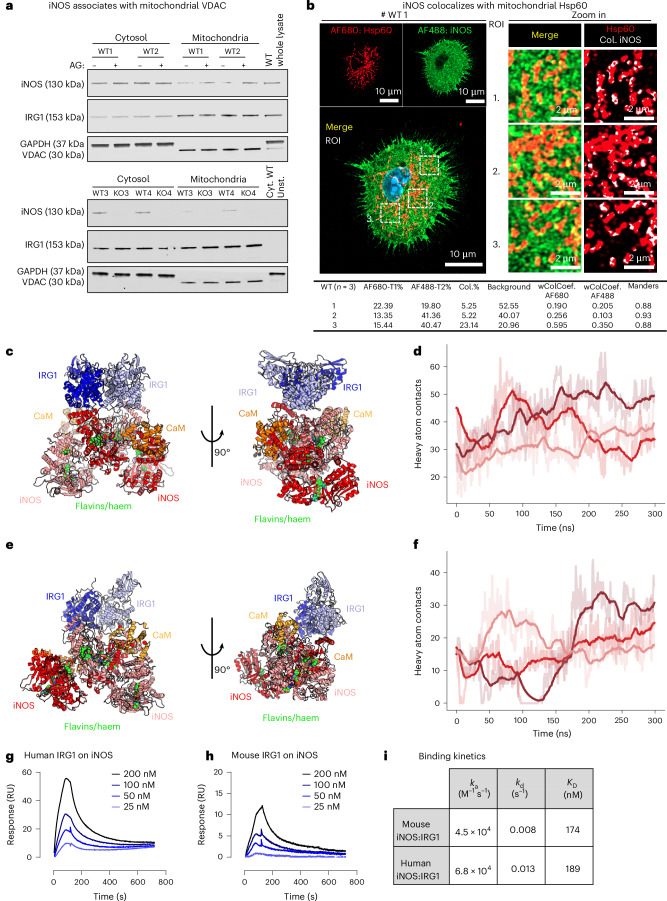
iNOS and IRG1 interact in macrophages. **a**, Representative western blot of two mitochondria isolation preparations (total *n* = 4 mice); 7 µg of protein for each cell compartment (mitochondria, cytosol or whole lysate) from 18 h LPS/IFNγ-stimulated murine BMDMs (WT iNOS BMDMs treated or not with AG and iNOS-KO BMDMs) was loaded into an SDS–PAGE and further probed with anti-iNOS, anti-IRG1, anti-GAPDH (cytosol control) and anti-VDAC (mitochondria control). **b**, Immunofluorescence of fixed BMDMs following 18 h LPS/IFNγ stimulation from WT and iNOS-KO mice after incubation with mouse anti-iNOS (AF488), anti-Hsp60 (AF680) and DAPI (blue). Single-channel images, as well as the superposition of channels (merge; yellow), are shown. Three regions of interest (ROIs) for each image were used for colocalization analysis. White indicates colocalization of iNOS and Hsp60 on Hsp60 staining (red). A table summarizing the mean of the colocalization factor and Manders’ coefficient from three ROIs for each animal (*n* = 3 mice) is also shown. **c**–**f**, Computational predictions of the (IRG1)_2_–(iNOS)_2_ heterotetramer using AlphaFold-Multimer for the murine (**c**) and human (**e**) heterotetramer, respectively. The two snapshots are related by a 90 °C rotation around the *z* axis. The predictions show a well-established interface between the two dimers. **c**, Predicted structure of the murine IRG1–iNOS heterotetramer (iNOS monomers are in red and pink and IRG1 monomers in blue and lavender) in the presence of calmodulin (orange) and haem and flavins (green). **d**, Molecular dynamics simulations of the murine IRG1–iNOS heterotetramer. The solid lines represent the number of heavy atom contacts between the (IRG1)_2_ and (iNOS)_2_ homodimers, and the different colours represent three different 300 ns replicas. The stable conformation observed over the 300 ns trajectory supports the reliability of the predicted protein–protein interface. **e**, Predicted structure of the human IRG1–iNOS heterotetramer (iNOS monomers are in red and pink and IRG1 monomers in blue and lavender) in the presence of calmodulin (orange) and haem and flavins (green). **f**, Molecular dynamics simulations of the human IRG1–iNOS heterotetramer. The solid lines represent the number of heavy atom contacts between the (IRG1)_2_ and (iNOS)_2_ homodimers, and the different colours represent three different 300 ns replicas. The stable conformation observed over the 300 ns trajectory supports the reliability of the predicted protein–protein interface. **g**–**i**, Binding kinetics of IRG1 and iNOS measured by surface plasma resonance. **g**, Multi-cycle kinetics analysis of human IRG1 binding to human iNOS. Surface plasma resonance sensograms show the average response curves from duplicate injections of human IRG1 over a human iNOS-immobilized CM5 sensor chip (25–200 nM). Fitted constants of *k*_a_ = 6.8 × 10^4^ M^−1^ s^−1^ and *k*_d_ = 0.013 s^−1^, which results in a *K*_D_ = 189 nM. **h**, Multi-cycle kinetics analysis of mouse IRG1 binding to mouse iNOS. Surface plasma resonance sensograms show the average response curves from duplicate injections of mouse IRG1 over a mouse iNOS-immobilized CM5 sensor chip (25–200 nM). Fitted constants of *k*_a_ = 4.5 × 10^4^ M^−1^ s^−1^ and *k*_d_ = 0.008 s^−1^, which results in a *K*_D_ = 174 nM. **i**, *k*_a_, association constant; *k*_d_, dissociation constant; *K*_D_, equilibrium constant of the IRG1–iNOS interaction for both human and mouse proteins; Cyt., cytosol; Col., colocalization; WColCoef., weighted colocalization coefficient; Unst., unstimulated. Source data

**Extended Data Fig. 5:**
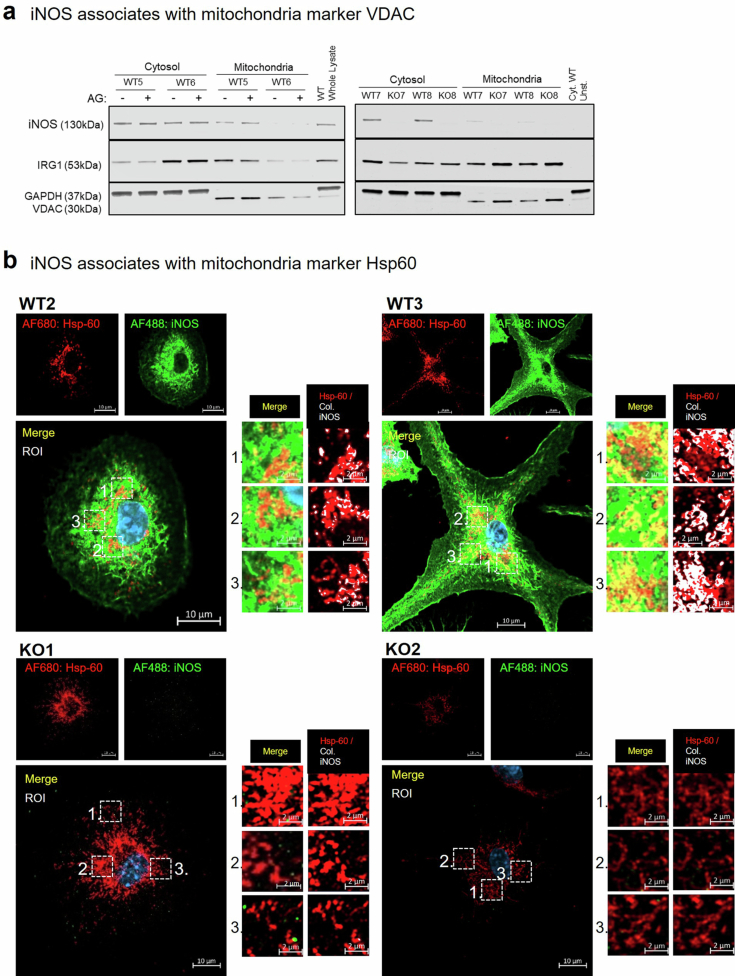
iNOS localises in mitochondria. **a**, Western blot of two mitochondria isolation (total n = 4 mice per group, 1 mitochondria isolation per mice, 2 shown by Western blot); 7 µg of protein for each cell compartment (mitochondria, cytosol or whole lysate) from 18 h LPS/IFNγ stimulated murine BMDMs (WT iNOS BMDMs treated or not with AG and KO iNOS BMDMs) was loaded into an SDS-PAGE and further probed with anti-iNOS, anti-IRG1, anti-GAPDH (cytosol control) and anti-VDAC (mitochondria control). **b**, Immunofluorescence of fixed BMDMs following 18 h LPS/IFNγ stimulation from WT and iNOS KO mice (n = 3 mice per group) after incubation with mouse anti-iNOS (AF488), anti-Hsp60 (AF680) and DAPI (blue). Single channel images as well as superposition of channels (Merge; yellow) are shown. 3 regions of interest (ROI) for each image were used for colocalisation analysis. White indicate colocalisation of iNOS and Hsp60 on Hsp60 staining (red). Source data

As iNOS is reported to be primarily located in the cytoplasm and IRG1 within the mitochondria^26,27^, we analysed isolated mitochondria from stimulated BMDMs. Both iNOS and IRG1 were found in mitochondria, and loss of iNOS or iNOS activity did not impact IRG1 location (Fig. 3a and Extended Data Fig. 5a). Immunofluorescence further supported this finding, showing iNOS colocalizing with the mitochondrial marker Hsp60 (Fig. 3b and Extended Data Fig. 5b).

**Extended Data Fig. 6:**
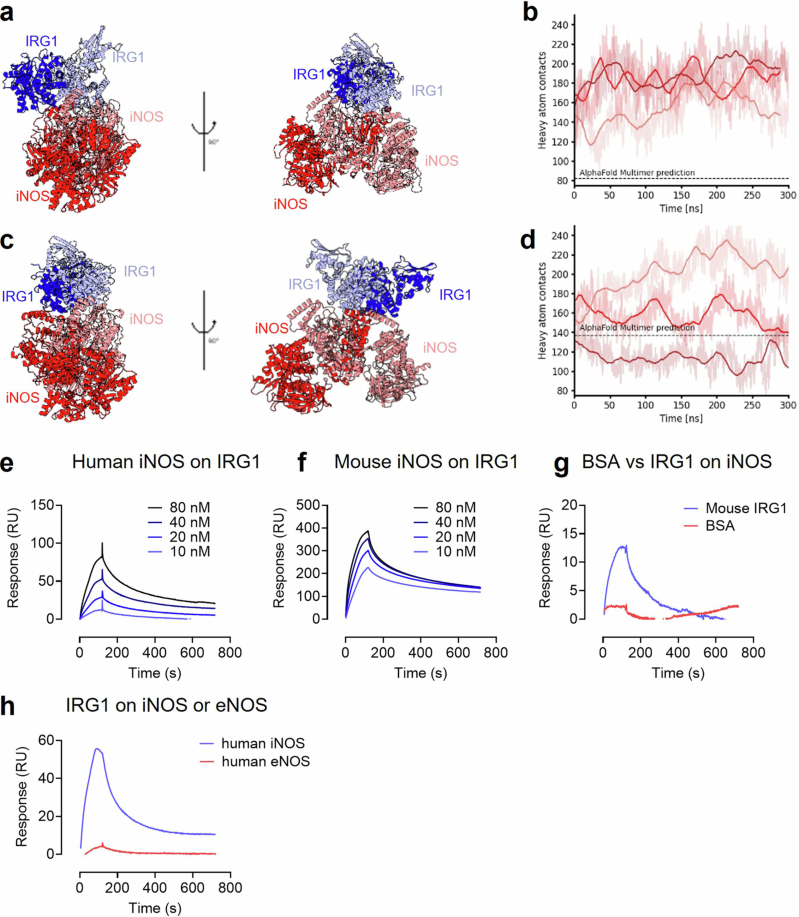
iNOS and IRG1 interact with strong affinity. **a**, Predicted structure of the murine IRG1 iNOS heterotetramer (IRG1 monomers are in red and pink and iNOS monomers in sky blue and blue) in the presence of calmodulin (orange and sand colour). **c**, Molecular dynamics simulations of the murine IRG1-iNOS heterotetramer. The solid lines represent the number of heavy atom contacts between the (IRG1)2 and (iNOS)2 homodimers, and the different colours represent three different 300 ns replicas. The stable conformation observed over the 300 ns trajectory supports the reliability of the predicted protein-protein interface. **b**, Predicted structure of the human IRG1-iNOS heterotetramer (IRG1 monomers are in red and pink and iNOS monomers in sky blue and blue) **d**, Molecular dynamics simulations of the human IRG1-iNOS heterotetramer in the presence of calmodulin. The solid lines represent the number of heavy atom contacts between the (IRG1)2 and (iNOS)2 homodimers, and the different colours represent three different 300 ns replicas. The stable conformation observed over the 300 ns trajectory supports the reliability of the predicted protein-protein interface. **e**, Representative multi-cycle kinetics curves for mouse iNOS on a mouse IRG1 immobilised chip (10 – 80 nM). **f**, Representative multi-cycle kinetics curves for human iNOS on a human IRG1 immobilised chip (10 – 80 nM). **g**, Comparison sensorgram of BSA injection (100 nM) on iNOS chip compared to IRG1 (100 nM). **h**, Average sensograms from injections of IRG1 (200 nM) over iNOS- and eNOS-immobilized chips show differential binding responses.

Next, we sought to predict a physical interaction between iNOS and IRG1 using computational modelling. As IRG1 (ref. ^28^) and iNOS^29^ are known to be homodimers, the structure of the mouse (IRG1)_2_–(iNOS)_2_ heterotetramer was modelled using AlphaFold (see Methods) (Fig. 3c–f and Extended Data Fig. 6a–d). The predicted structure showed a clear interaction between (IRG1)_2_ and (iNOS)_2_, with the dimers oriented perpendicularly to each other and interacting at a binding groove involving all four monomers (Extended Data Fig. 6a). The subunits exhibited high similarity to the deposited crystal structures: the CA-only root mean square deviation between the published crystal structures (PDB 2NOD for iNOS and PDB 6R6T for IRG1) and the corresponding protein chains in the predicted multimer structures were all <1 Å. Furthermore, the structure of iNOS was in the input state, characterized by the close proximity between the flavins in the dimer^30^. As AlphaFold predictions are known to be good predictors of protein–protein interactions (PPIs)^31^, we evaluated the stability of the predicted interface by carrying out molecular dynamics simulations of the heterotetramer. We observed that the binding interface remained stable throughout three replicate simulations of 300 ns each (Extended Data Fig. 6b). The monomers tended to relax towards a more symmetrical conformation (Supplementary Videos 1–3), and the number of contacts between the (IRG1)_2_ and (iNOS)_2_ dimers tended to increase, rather than decrease, throughout the simulation. We further sought to validate the interaction by performing MM/GBSA free energy calculations (see Methods), observing a free energy of binding of −152.3 ± 0.2 kcal mol^−1^. These results support a model in which the IRG1 and iNOS species interact directly in solution. To further understand the relevance of the study in the context of human health, we set out to extend the computational modelling to the human heterotetramer. AlphaFold predictions reveal a binding pose similar to the mouse analogue^32^ (Extended Data Fig. 6c,d). Molecular dynamics simulations and free energy calculations suggest the stability of the pose (Extended Data Fig. 6c,d and Supplementary Videos 4–6). We repeated the structural modelling and molecular dynamics experiments in the presence of iNOS cofactors calmodulin, flavin adenine dinucleotide and flavin mononucleotide, for both murine and human heterotetramers. The predicted structures closely resembled the poses without calmodulin, although with a smaller rotation between the IRG1 dimer and the iNOS-calmodulin dimer (45° instead of 90°) (Fig. 3c–f).

We next used surface plasma resonance to validate the interaction and assess binding kinetics between IRG1 and iNOS in both mouse and human models (see Methods). Although the constants indicated a moderate association and dissociation between iNOS and IRG1, the resultant low equilibrium dissociation constant demonstrated that the interaction between iNOS and IRG1 is stable and has a high affinity (Fig. 3g–i and Extended Data Fig. 6e–h). This interaction appears specific to iNOS, as endothelial NOS (eNOS) did not bind IRG1 (Extended Data Fig. 6h). Furthermore, similar kinetics values between the human and the mouse model suggest that this interaction is evolutionarily conserved. These results, together with the computational modelling, support the hypothesis that IRG1 and iNOS interact in both human and animal models.

**Fig. 4:**
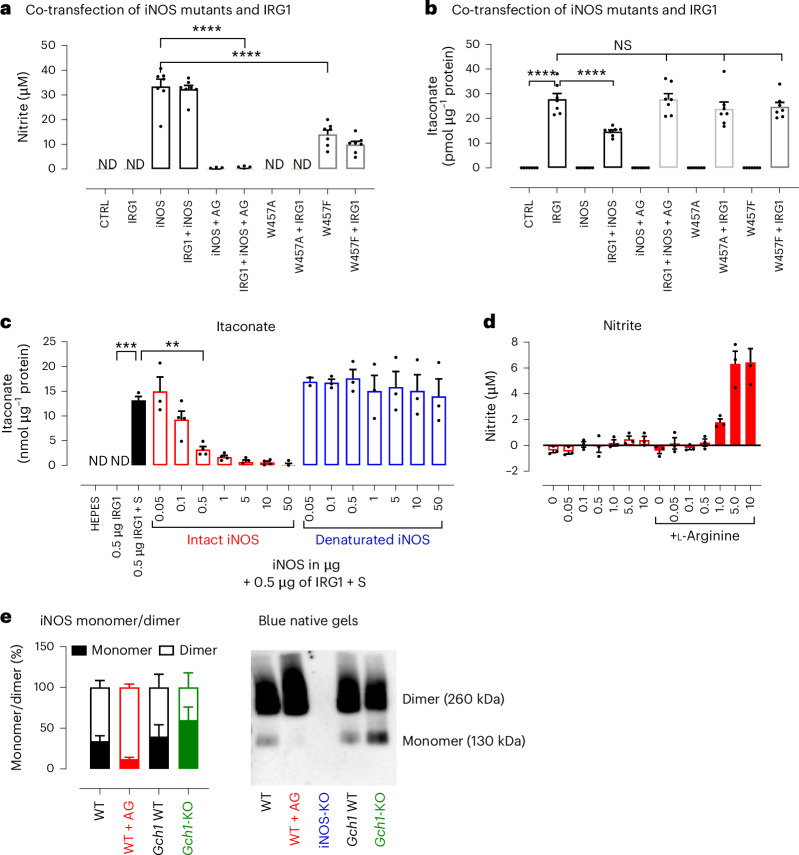
iNOS inhibition of IRG1 relies on BH4 but does not require l-arginine. Following transfection of HEK cells with iNOS cDNAs (WT and mutants W457A and W457F), alone or in combination with IRG1 cDNA, the ability of each iNOS to regulate IRG1 was tested. **a**,**b**, Nitrite production by Griess assay (**a**) and itaconate in cell pellets by HPLC (**b**) were measured in *n* = 7 independent experiments. Data are presented as mean values; error bars, s.e.m. Statistical differences were calculated using one-way ANOVA with Dunnett’s multiple comparisons test against the IRG1 condition for itaconate measurement and against the iNOS condition for nitrite measurement. Protein levels are shown in Extended Data Fig. 8. **c**, A 0.5 µg aliquot of IRG1 was incubated with 25 µM of *cis*-aconitate (substrate (S)) with increasing concentration of intact (*n* = 4 independent experiments) or denaturated (*n* = 3 independent experiments) iNOS in the absence of arginine for 18 h at 37 °C. Itaconate levels were determined by HPLC using an itaconate standard to normalize the data. Data are expressed as mean values; error bars, s.e.m. Statistical differences were calculated using one-way ANOVA with Dunnett’s multiple comparisons test against the 0.5 µg IRG1 + S condition. **d**, Nitrite levels of intact IRG1 in the absence or presence of l-arginine and other iNOS cofactors (BH4, NADPH, flavin adenine dinucleotide (FAD), flavin mononucleotide (FMN)). Data are represented as mean values of *n* = 3 independent experiments; error bars, s.e.m. **e**, iNOS native form in M(LPS/IFNγ) stimulated for 18 h in WT, iNOS-deficient, *Gch1* WT and *Gch1*-KO BMDMs using blue native gels and non-denaturing conditions; *n* = 4 mice for the *Gch1*-KO model and *n* = 5 mice for the iNOS-KO model (details in Methods). Statistical significance is indicated as ****P < 0.0001; ***P < 0.001; **P < 0.005. Source data

We next interrogated further the roles of the iNOS cofactor, BH4, and the substrate for NO production, l-arginine, in the iNOS-mediated inhibition of IRG1. First, we generated two iNOS mutants targeting the W457 residue (W457A and W457F) that have a crucial role in BH4 binding^33–35^ (Fig. 4a,b and Extended Data Fig. 8a). When W457 is replaced with alanine (W457A), BH4 binding is compromised, preventing production of NO^33–35^. However, replacing W457 with phenylalanine (W457F) reduces but does not abolish NO production, owing to partial retention of BH4 binding by the aromatic ring. Following co-transfection of IRG1 and iNOS mutants, we confirmed that although W457A led to complete inhibition of NO production, W457F maintained nitrite levels at ~50% of WT iNOS, but neither W457A nor W457F were able to inhibit itaconate production, indicating that BH4 binding to iNOS is crucial for IRG1 inhibition, independent of NO production. Second, to understand whether iNOS requires l-arginine and subsequent production of NO to inhibit itaconate production, increasing concentrations of either readily active iNOS (commercially obtained and purified with BH4) or denatured iNOS (obtained by heat inactivation of iNOS at 95 °C) were added to 0.5 µg of purified IRG1 in the absence of l-arginine and other cofactors (Fig. 4c and Extended Data Fig. 8b). Strikingly, the addition of intact iNOS (at a 1:1 iNOS-to-IRG1 ratio) was sufficient to inhibit >75% of itaconate production. By contrast, addition of denatured iNOS (up to a 100:1 ratio) had no effect on itaconate levels. These observations indicate that although BH4 is crucial for iNOS to inhibit IRG1 activity, its ability to produce NO is not essential (Fig. 4d).

**Extended Data Fig. 7:**
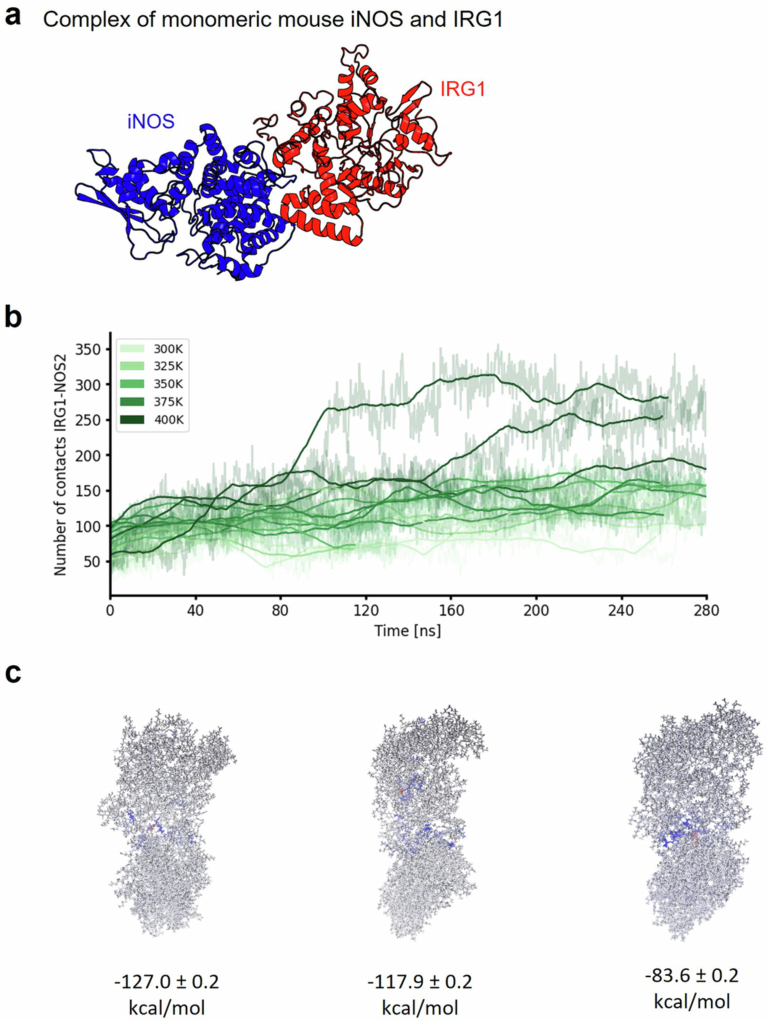
Computational modelling of the interaction between monomers of mouse IRG1 and iNOS. **a**, AlphaFold Multimer v2.3 prediction of the IRG1-iNOS dimer (IRG1 monomer with iNOS monomer). **b**, Molecular dynamics simulations at different temperatures showing that the interaction between the units is stable. **c**, MM/GBSA free energy calculations of three snapshots of the molecular dynamics simulations.

**Extended Data Fig. 8:**
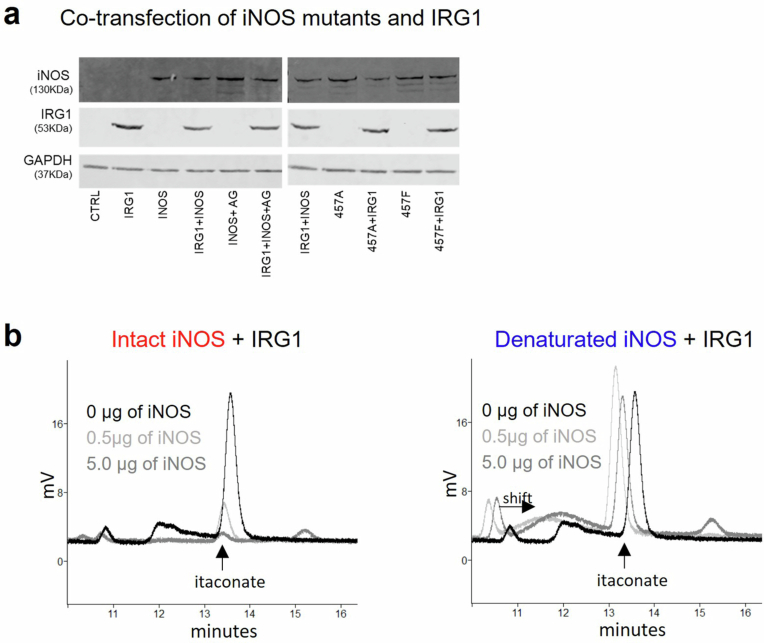
iNOS inhibition of IRG1 relies on BH4 but does not require l-arginine. **a**, Representative Western blot of n = 3 independent experiment following transfection of HEKs cells with iNOS cDNAs (WT and mutants W457A and W457F), alone or in combination with IRG1 cDNA. GAPDH was used as a loading control. **b**, Example of HPLC chromatograph measuring itaconate produced by IRG1 for 18 h at 37 °C in HEPES buffer and incubated with intact iNOS or denatured iNOS. Source data

Given that we previously showed that inhibiting iNOS production of NO with AG restores itaconate levels, despite the presence of BH4 (Fig. 2a–c), we reasoned that a change in iNOS conformation rather than the ability to inhibit NO by AG could explain this contradictory finding. AG inhibits iNOS by targeting multiple steps during iNOS catalytic activity, creating a covalent adduct between iNOS and BH4, which affects the ability of iNOS to undergo conformational changes that are required to produce NO and ‘blocks’ it in a dimeric form^36,37^. Other studies have shown that iNOS shifts between monomeric and dimeric forms depending on BH4, which acts as a stabilizer of the iNOS conformation; in its presence, iNOS tends to be more dimeric, whereas in its absence, iNOS exhibits a higher proportion of the monomeric form^10,38^. Additionally, although iNOS-W457A and iNOS-W457F dimerize, their capacity to displace haem-bound imidazole by arginine is prevented, suggesting a ‘freezing’ of iNOS conformation that is unable to produce NO^35^. Using blue native gel electrophoresis, we demonstrated that iNOS from WT macrophages was mainly composed of dimeric iNOS, with a smaller proportion of monomeric form. In the absence of BH4, in *Gch1*-KO macrophages, the proportion of iNOS monomer was increased, whereas in the presence of AG (WT + AG), iNOS was almost entirely dimeric (Fig. 4e). These results are consistent with our computational modelling, in which the interaction energy between the dimers is higher (~−150 kcal mol^−1^) than in a complex of monomeric iNOS and IRG1 (~−110 kcal mol^−1^) (Extended Data Fig. 7). The dynamic nature of iNOS was also exemplified in the conformational diversity of AlphaFold multimer predictions discussed previously and suggests that itaconate inhibition relies on iNOS being in a dynamic dimer able to undergo conformational changes.

## Discussion

Our findings reveal an immunoregulatory role for iNOS in modulating itaconate levels through direct interaction with IRG1, independent of its classical NO synthase activity, but dependent on BH4 stabilization and subsequent conformational changes. These findings provide insight into the role of iNOS and BH4 in modulating itaconate in health and disease. We initially observed a striking difference between intracellular and extracellular levels of itaconate, with tenfold to 100-fold more itaconate released over time in WT macrophages. This compartmental difference is consistent with previous reports^11,39^. Notably, our study shows that inhibiting iNOS led to more than twofold more itaconate being released by macrophages, providing evidence that targeting the paracrine effects of itaconate and the IRG1–iNOS signalling axis could be a potential therapeutic target. Additionally, we demonstrate that in the absence of iNOS, IRG1 interacts with redox, immune and glycolytic partners, including pyruvate kinase, fructose-bisphosphate aldolase A (*Aldoa*), interleukin-1 alpha (*Il1a*) and superoxide dismutase (*Sod1*). This implies that iNOS inhibits the broader immunometabolic roles of IRG1 by ‘sequestering’ it away from alternative partners. As recent studies have stressed the difference in action of 4-octyl itaconate and dimethyl itaconate to modulate inflammation in comparison to native itaconate, increasing its endogenous level and targeting the IRG1 interactome by preventing iNOS inhibition could be an important alternative strategy^25,40^.

Our study also highlights the wide range of itaconate levels reported in the literature, from nmol g^−1^ to mM (Extended Data Table 2). We show here itaconate levels in the pmol µg^−1^ of protein range, consistent with other publications; however, such variability might arise from differences in quantification and estimation methods, which should be addressed within the field to enable better comparison across studies.

Finally, our findings add to growing evidence of PPIs as a key regulatory mechanism to target therapeutically^41^. Although PPIs involving eNOS and neuronal NOS are well established, we show here that iNOS also engages in functional PPIs, similarly to its inhibitory interaction with aldose reductase^42–44^. Although many studies have used point mutagenesis to dissect NOS function and interactions, the NO-generating and IRG1-binding functions of iNOS may not be separable through a mutagenesis strategy, as the requirement for a single iNOS residue being essential for the iNOS–IRG1 interaction remains speculative. We have demonstrated that the W457F-iNOS mutant can produce NO but fails to inhibit IRG1 owing to impaired BH4 binding. Generating iNOS mutants that are unable to produce NO yet continue to inhibit IRG1 activity will help to provide further insights into the molecular mechanisms by which iNOS regulates IRG1 activity. Most likely, the iNOS–IRG1 interaction may be driven by conformational changes that involve more than a single residue.

These results emphasize the broader role of iNOS in cellular metabolism and immune regulation in addition to NO production. Targeting the iNOS–IRG1–itaconate axis could be achieved by designing inhibitor peptides or small molecules directed to the iNOS–IRG1 interfaces, preventing their direct contact based on further structural or mutational analyses. The new insights from this study provide a rational framework to guide the development of such strategies.

## Methods

### Animal details

All animal procedures were approved and carried out in accordance with the University of Oxford Ethical Committee and the UK Home Office Animals (Scientific Procedures) Act 1986 and conformed with Directive 2010/63/EU of the European Parliament. We used *Nos2*^−/−^ (*Nos2*^*tm1Lau*^) (iNOS-KO) and WT C57BL6/J mice purchased from The Jackson Laboratory (stock no. 002609). A conditional KO (floxed) of the *Gch1* allele using the Cre/loxP strategy was generated as previously described, producing *Gch1* WT (*Gch1*^fl/fl^; containing the gene encoding BH4) and *Gch1*-KO (*Gch1*^fl/fl^; *Tie2-*Cre; with excision of the gene encoding BH4) mice. Experiments were performed using bone marrow isolated from 10–16-week-old adult male and female mice for all genotypes^1,10,45^.

### Isolation and stimulation of BMDMs

BMDMs were obtained and cultured as described in our published protocol^13,46^. In brief, after bone marrow cell isolation, cells were plated in non-tissue-culture-treated plastic at 1 × 10^6^ cells per well of a six-well plate for time course experiments or 3 × 10^6^ cells per 10 cm dish for IP experiments using DMEM:F12 (ThermoFisher Scientific) supplemented with penicillin (100 U ml^−1^), streptomycin (100 ng ml^−1^; Sigma-Aldrich), ultra-low-endotoxin FBS (5% (v/v); Biowest) and l-glutamine (5 mmol l^−1^; Sigma-Aldrich). Recombinant macrophage colony-stimulating factor (M-CSF) protein (Peprotech) and recombinant granulocyte M-CSF (GM-CSF) protein (Peprotech) were added at day 0, day 5, day 6 and day 7 to ensure macrophage differentiation (see published protocol for concentration). Cells were then stimulated with IFNγ (10 ng ml^−1^; Peprotech) and LPS (100 ng ml^−1^; Sigma-Aldrich) in 2% (v/v) in low-endotoxin Biowest FBS-supplemented DMEM:F12. If specified, cells were treated with 1 mM aminoguanidine hydrochloride (Sigma-Aldrich, 396494) at the same time as LPS and IFNγ. Cells were then collected at different time points.

### Culturing HEK-293T cells

HEK-293T (HEK) cells were obtained from European Collection of Authenticated Cell Cultures (ECACC; Merck, cat. no. 12022001) and passaged routinely in DMEM supplemented with penicillin, streptomycin and 10% (v/v) FBS (Sigma-Aldrich, F7524).

### Culturing hMDMs

PBMCs were obtained from anonymous healthy donors (sex unknown). Informed consent and ethical approvals were obtained from the NHS Blood and Transplant service (UK). PBMCs were isolated from leucocyte cones after gently layering blood over Histopaque (Sigma-Aldrich). Following density centrifugation, the PBMC layer was carefully collected and washed repeatedly with PBS and centrifuged. CD14^+^ monocytes were then isolated by positive selection using magnetic beads conjugated to anti-CD14 antibody (EasySep Human Monocyte Isolation Kit, Stemcell Technologies). Once isolated, 1 x 10^6^ cells were plated in a six-well plate in RPMI 1640 medium supplemented with penicillin (100 U ml^−1^), streptomycin (100 ng ml^−1^; Sigma-Aldrich), FBS (10% (v/v); Sigma-Aldrich), l-glutamine (5 mmol l^−1^; Sigma-Aldrich) and 100 ng ml^−1^ of human recombinant M-CSF (Peprotech) (day 0). At day 5, 50 ng ml^−1^ of human recombinant GM-CSF (Peprotech) was added, followed by 100 ng ml^−1^ on day 6. On day 7, cells were stimulated with IFNγ (20 ng ml^−1^; Peprotech) and LPS (100 ng ml^−1^; Sigma-Aldrich) in 2% (v/v) FBS-supplemented RPMI with 100 ng ml^−1^ of GM-CSF.

### Human bone marrow organoids culture and stimulation

KOLF2.1J human iPS cells were kindly offered by the Induced Pluripotent Stem Cell Facility from the NDM–Centre for Human Genetics at the University of Oxford. The iPS cells were differentiated into bone marrow organoids following a previously published protocol^21^ using a variety of cytokines, chemokines and growth factors^21,47^. In brief, cells were first allowed to form iPS cell aggregates and undergo mesodermal induction (days 0–3) before committing to vascular and haematopoietic lineages (days 3–5). These aggregates were then embedded into a mixture of collagen-Matrigel hydrogels, allowing vascular sprouting (days 5–12). At day 12, each sprout was collected and added one by one into 96-well ultra-low attachment plates. At day 16, bone marrow organoids were formed containing haematopoietic, endothelial and stromal cells, organized in 3D and able to generate myeloid cell types. These newly formed organoids were then stimulated for 24 h with 20 ng ml^−1^ of IFNγ, 100 ng ml^−1^ LPS or LPS and IFNγ. To assess gene expression and intracellular itaconate levels, four organoids (=four wells) were pooled together, counting as a representative of *n* = 1 biological replicate. Organoids were then centrifuged gently at 1,000*g* for 5 min and digested with 2.5 mg ml^−1^ of Collagenase II (17101-015, Gibco) and 2% BSA in DMEM for 15 min at 37 °C. This incubation was repeated until organoids formed a single-cell suspension. Cells were then spun at 500*g* for 5 min and the pellet obtained was directly processed for real-time qPCR with reverse transcription analysis or itaconate measurement by mass spectrometry.

### Real-time qPCR

Total RNA was extracted from cells using the Qiagen RNeasy Mini Kit. RNA purity and quantity were then determined using an ND-1000 Nanodrop spectrophotometer and converted to cDNA using the Qiagen QuantiTect Reverse Transcription Kit. Using the QuantStudio 6 Flex Real-Time PCR System, 5 ng of cDNA was used for each qPCR reaction in a 384-well plate alongside TaqMan Fast Advanced Master Mix and TaqMan assay primers specific of our genes of interest (respectively; *Acod1* (Hs00985781_m1), *Nos2* (Hs01075529_m1), *Gch*1 (Hs00609198_m1)) or internal control (either *18S* (4333760T) or *GAPDH*(4326317E)). Gene expression was then assessed using the 2^−ΔΔ^^*Ct*^ method, where ΔΔ*Ct* is the fold change for each condition relative to unstimulated cells following normalization using an internal control.

### Fugene-HD transfection and stimulation

HEK cells were plated in a six-well plate at 1 × 10^6^ cells in 2 ml. At 60–70% confluence, cells were transfected at a ratio of 1:3 of plasmid DNA:Fugene-HD. In brief, 3 µl of Fugene-HD was added to 100 µl serum-free DMEM, mixed well and left for 10 min. Then, 1 µg of plasmid DNA (either *Nos2* (NM_010927) mouse tagged ORF clone or WT or mutated *Acod1* (NM_008392) mouse tagged ORF clone) was then added to the Fugene-HD solution. Following incubation for 10 min at room temperature (20°C to 22°C), 100 µl of transfection mix was added to the cell media. The cells were collected 24 h later.

Transfected cells were treated 1 h following transient transfection with 50 µM of NOC12 or NOC18 (EMD Millipore, 487955 or Sigma-Aldrich, 487957, respectively), 10 µM of 1400W dihydrochloride (Sigma-Aldrich, W4262), 1 mM aminoguanidine hydrochloride or 100 µM of SIN-1 chloride (Tocris, 5245).

### Mutagenesis of IRG1

Acod1 (NM_008392) mouse tagged ORF clone was subjected to different mutations, using the QuickChange Lightning Site-Directed Mutagenesis kit if only one mutation was required (C184A, C340A, C387A, C432A, C452A, T319F) and the QuickChange Lightning Multi Site-Directed Mutagenesis kit if more than one mutation was required (for example, ALA5). Primers used were:

C184A(MF)cys184ala-IRG15′-CTCAGCTTGACAAAGGCCCGCGAGGCATTGGCT-3′

(MR)cys184ala-IRG15′-AGCCAATGCCTCGCGGGCCTTTGTCAAGCTGAG-3′

C340A (MF)cys340ala-IRG15′-TTCCAGTATGTGGCCGCTGCCTCGCTGCTCGAC-3′

(MR)cys340ala-IRG15′-GTCGAGCAGCGAGGCAGCGGCCACATACTGGAA-3′

C387A(MF)cys387ala-IRG15′-TTCGACACGCTATACGCTGAAATAAGCATCACT-3′

(MR)cys387ala-IRG15′-AGTGATGCTTATTTCAGCGTATAGCGTGTCGAA-3′

C432A (MF)cys432ala-IRG15′-GCCTCAAAGATGCTAGCCAGGGACACGGTGGAA-3′

(MR)cys432ala-IRG15′-TTCCACCGTGTCCCTGGCTAGCATCTTTGAGGC-3′

C452A (MF)cys452ala-IRG15′-GAAGACCTAGAAGACGCCTCTGTGCTAACCAGA-3′

(MR)cys452ala-IRG15′-TCTGGTTAGCACAGAGGCGTCTTCTAGGTCTTC-3′

Validation of mutants was performed using the Source BioScience sequencing platform (see Extended Data Fig. 3g).

### Mutagenesis of iNOS

The *Nos2* (NM_010927) mouse tagged ORF clone was subjected to different mutations (W457A and W457F) using the QuickChange Lightning Site-Directed Mutagenesis kit. Primers used were:

W457A (MF)-iNOS: 5′-GAGGGACCAGCGCAATCCAGTCTGCCGGG-3′

(MR)-iNOS: 5′-CCCGGCAGACTGGATTGCGCTGGTCCCTC-3′

W457F (MF)-iNOS: 5′-GGAGGGACCAGGAAAATCCAGTCTGCCGGGC-3′

(MR)-iNOS: 5′-GCCCGGCAGACTGGATTTTCCTGGTCCCTCC-3′

Validation of mutants was performed using the Source BioScience sequencing platform.

### Dihydroethidium HPLC

Cultured macrophages were incubated with 25 µM dihydroethidium (Invitrogen) for 15 min at 37 °C and were protected from light before collection. Cell pellets were lysed in ice-cold methanol, and protein was removed by acid precipitation with 0.1 M HCl. Separation of dihydroethidium and its oxidized products, 2-hydroxyethidium and ethidium, was performed using a gradient HPLC system (Jasco) with an ODS3 reverse phase column (250 mm, 4.5 mm; Hichrom) and quantified using a fluorescence detector set at 510 nm (excitation) and 595 nm (emission). A linear gradient was applied from mobile phase A (0.1% (w/v) trifluoroacetic acid) to mobile phase B (0.085% (w/v) trifluoroacetic acid in acetonitrile), over 23 min (30–50% (v/v) acetonitrile).

### H_2_O_2_ measurement

Media from transfected HEK cells, seeded at 1 x 10^6^ cells in six-well plates, were centrifuged to remove debris at 500*g* for 5 min at 4 °C and frozen directly. The H_2_O_2_ concentration was then measured by inserting an amperometric H_2_O_2_ microsensor electrode connected to a free radical analyser system (WPI) into a PBS solution in which 100 µl of thawed media was added. The output current corresponding to the H_2_O_2_ concentration detected was recorded and analysed using LabTrax 2 software. Sample concentrations were determined using a known H_2_O_2_ standard curve.

### Western blotting using SDS–PAGE

Cell lysates were prepared by homogenization in ice-cold CellLytic M buffer (Sigma-Aldrich) containing protease inhibitor cocktail (Roche Applied Science). Lysates were centrifuged at 17,000*g* for 10 min at 4 °C, and samples were prepared using NuPAGE LDS sample buffer (Invitrogen) with the addition of 10% dithiothreitol (DTT). Then, 5–10 µg of protein was loaded per well in a 4–12% Bis-Tris NuPAGE gel (Thermofisher) and transferred to a nitrocellulose membrane. Anti-iNOS (Abcam, ab49999; diluted at 1:1,000), anti-IRG1 (Abcam, ab222411; diluted at 1:1,000) and anti-GAPDH (Merck, MAB374; diluted at 1:1,000) were then coupled with Licor antibodies (IRDye680RD goat anti-mouse and IRDye488RD goat anti-rabbit; both diluted at 1:25,000). Anti-iNOS (BD Biosciences, 610431; diluted at 1:1,000) was coupled to anti-mouse IgG (H + L) and HRP conjugate (Promega, W4028; diluted at 1:25,000). Proteins were detected using the ChemiDoc Imaging system (Bio-Rad) following Amersham ECL Select western blotting detection reagent (Cytiva, RPN2235).

### Nitrite measurement

Nitrite accumulation was measured in the cell culture medium of the samples using the Griess assay with colourimetric detection in 96-well plates. Cell culture supernatants were mixed 1:1 with the Griess reagent (Sigma-Aldrich) and quantified by comparison to a sodium nitrite (Sigma-Aldrich) standard curve produced in tissue culture media at 550 nm.

### Itaconate measurement by HPLC

Intracellular itaconic acid levels were measured using HPLC^48–51^. Pellets from 1 x 10^6^ macrophage cells were resuspended in PBS at pH 7.4 and lysed by three freeze–thaw cycles. After centrifugation at 17,000*g* for 15 min at 4 °C, debris-free lysates were transferred to new tubes. Proteins were then precipitated by the addition of 10% (v/v) of 2 M perchloric acid and centrifuged at 17,000*g* for 15 min at 4 °C. Cell media was centrifuged at 500*g* for 5 min at 4 °C before the addition of 10% (v/v) of 2 M perchloric acid and centrifuged at 17,000*g* for 15 min at 4 °C. Samples were injected onto a 250 mm ACE C18 column (Hichrom), and itaconate was quantified using UV detection at 210 nm. HPLC separation was performed using a mobile phase comprising of 2.5% (v/v) acetonitrile and 0.1% (v/v) phosphoric acid (all ultrapure electrochemical HPLC grade), at a flow rate of 1.0 ml min^−1^. Quantification of itaconic acid was made by comparison with pure itaconic acid (Sigma-Aldrich, I29204) standard range from 0.50 μM to 500 μM in PBS or cell media. Final results were normalized using each sample’s protein concentration, determined by the bicinchoninic acid protein assay. Extracellular itaconate levels were calculated by comparing the itaconate concentration in cell media samples to a standard prepared in the same media, then multiplying by the total volume exposed to the cells and normalizing to the protein content.

### Itaconate measurement by ion chromatography coupled to mass spectrometry

Human iPS cell pellets were lysed in ice-cold methanol and then spun at 17,000*g* for 30 min. The supernatant containing intracellular itaconate was then measured using ion chromatography coupled to mass spectrometry as detailed previously^52^. DNA was further assessed for normalization.

### Purification of mouse IRG1

The IRG1 sequence from Acod1 (NM_008392) mouse tagged ORF clone was expressed in the pOPINEneo-3C-Strep2-His8 vector in which IRG1 was tagged at the carboxy terminus with a Strep-Tag II. The plasmid was transiently expressed in HEK cells (expi293, Thermofisher Scientific) as previously described^53^. Cells were then lysed in 50 mM Tris, pH 7.5, 500 mM NaCl, 30 mM imidazole, 0.2% Tween, protease inhibitors and DNAse I, using a cell disruptor. Debris were removed by centrifugation at 30,000*g* for 30 min at 4 °C. The transfected protein was then purified from cleared lysates using the StrepTactin purification protocol and reagents from IBA Lifesciences. Protein was concentrated in GF buffer (10 mM HEPES, 150 mM NaCl, 0.1 mM TCEP, 10% (v/v) glycerol) as previously proposed^28^. A western blot and Coomassie gel were performed after each purification to assess the quality of the purification process.

### IRG1 enzymatic activity

A 2.5 µg aliquot of purified IRG1 in GF buffer was added to 150 µl of 0.2 M sodium phosphate buffer at pH 6.5 containing 15 µM *cis*-aconitate for 4 h and incubated at 37 °C. Drugs (1 mM of sodium nitrite, 1 mM SIN-1, 1 mM NOC12 and NOC18, 1 mM GSNO (+10 mM of GSH or GSSG), 1 mM H_2_O_2_, 1 mM DTT, 50 or 500 µM of citrulline and 50 or 500 µM of arginine) were added at the same time as purified IRG1. Itaconate was then determined by boiling samples at 95 °C, centrifuging at 17,000*g* for 5 min and injecting 100 µl onto the HPLC column.

To assess interaction with iNOS, 0.5 µg of purified IRG1 was added to 200 µl of HEPES pH 7.4 with 25 µM of *cis*-aconitate overnight and incubated at 37 °C with different concentrations of mouse recombinant active iNOS from Cayman Chemical (60864; purified in 50 mM HEPES, pH 7.4, with 10% glycerol, 8 µM BH4, 2% protease inhibitor cocktail (EDTA free) and 0.05% nuclease), ranging from 0.05–50 µg. Inactivated iNOS was obtained by boiling the protein at 95 °C for 10 min. Respective protein concentrations of iNOS and IRG1 were obtained using the bicinchoninic acid assay.

### Co-IP of IRG1 and analysis by mass spectrometry

BMDMs were grown at a density of 1 x 10^7^ cells in a T75 non-treated flask. Cell pellets were then lysed in NP-40 buffer (50 mM Tris base, 0.5% (v/v) NP-40, 150 mM NaCl, 20 mM MgCl_2_ in distilled water) containing phosphoSTOP and protease inhibitor cocktail. Lysates were incubated overnight at 4 °C with Protein A-dynabeads (Invitrogen), pre-washed and pre-incubated with target (anti-IRG1 (ab222411)) and control antibodies (rabbit IgG isotype control; Cell Signaling Technology, 3900S). After washing beads from the cell lysate, the target protein was either eluted in 2.5× Laemmli loading buffer at 95 °C for western blot analysis or directly sent to the mass spectrometry facility. Samples were processed by on-bead digestion (SMART digestion using SMART Digest Kit Soluble Trypsin). Peptides obtained were then resuspended in 5% (v/v) formic acid and 5% (v/v) dimethylsulfoxide and then trapped on an Acclaim PepMap 100 C18 HPLC Column (Thermo Fisher, PepMapC18; 300 µm × 5 mm, 5 µm particle size) using solvent A (0.1% (v/v) formic acid in water) at a pressure of 60 bar and separated on an Ultimate 3000 UHPLC system (Thermo Fisher Scientific) coupled to a QExactive mass spectrometer (Thermo Fisher Scientific). The peptides were separated on an EASY-Spray PepMap RSLC column (75 µm i.d. × 2 µm × 50 mm, 100 Å; Thermo Fisher) and then electrosprayed directly into a QExactive mass spectrometer (Thermo Fisher Scientific) through an EASY-Spray nano-electrospray ion source (Thermo Fisher Scientific) using a linear gradient (length, 60 min; 5% to 35% solvent B (0.1% (v/v) formic acid in acetonitrile), with a flow rate of 250 nl min^−1^). The raw data were acquired on the mass spectrometer in data-dependent mode. Full scan MS spectra were acquired in the Orbitrap (scan range, 380–1,800 *m*/*z*; resolution, 70,000; AGC target, 3 × 10^6^; maximum injection time, 100 ms). After the mass spectrometry scans, the 15 most intense peaks were selected for HCD fragmentation at 28% of normalized collision energy. HCD spectra were also acquired in the Orbitrap (resolution, 17,500; AGC target, 1 × 10^5^; maximum injection time, 128 ms) with a first fixed mass at 100 *m*/*z*.

The raw data files generated were processed using MaxQuant (v.1.6.17.0) and integrated with the Andromeda search engine. For protein group identification, peak lists were searched against the human database (UPR_MusMusculus_UP00000589_10090_.fasta) as well as a list of common contaminants by Andromeda. Trypsin with a maximum number of missed cleavages of two was chosen. Oxidation and deamidation were used as variable modifications, while carbamidomethylation was set as a fixed modification. Protein and PSM false discovery rate were set at 0.01. Match between runs was applied. Data were uploaded to PRIDE (project accession, PXD048712). Results obtained by mass spectrometry were analysed by median normalization using Perseus and are displayed as log_2_ values.

### Isolation of mitochondria

Murine BMDMs were grown at a density of 750,000 cells in 10 cm Petri dishes and stimulated for 18 h with 100 ng ml^−1^ LPS and 10 ng ml^−1^ IFNγ, in the presence or absence of 1 mM AG. Mitochondria and cytosol were then isolated using the standard procedure of the Qproteome Mitochondria Isolation Kit (QIAGEN). In brief, cells were collected and washed using a 0.9% sodium chloride solution before being lysed and mechanically disrupted using a blunt needle and syringe. Following a series of centrifugation and washes, the mitochondria and cytosol fraction were separated, and 7 µg of protein from each compartment was analysed using SDS–PAGE with GAPDH as a cytosolic control (Merck, MAB374; diluted at 1:1,000) and VDAC as a mitochondria control (Cell Signaling Technology, 4661S; diluted at 1:1,000); proteins of interest were studied using anti-iNOS (Abcam, ab49999; diluted at 1:1,000) and anti-IRG1 (Abcam; ab222411; diluted at 1:1,000). Each primary antibody was then coupled with Licor antibodies (IRDye680RD goat anti-mouse and IRDye488RD goat anti-rabbit; both diluted at 1:25,000). The same threshold (brightness, contrast, opacity and filters) was applied during analysis between the target and its respective loading control using Licor to ensure accurate comparison of protein levels.

### Immunofluorescence staining and Airyscan imaging

Following cell culture of BMDMs in 10 cm dishes, cells were gently detached at day 6 and plated at 300,000 cells on coverslips in a 12-well plate in DMEM:F12 supplemented with 2% (v/v) FBS. After cell adherence, BMDMs were stimulated with LPS/IFNγ overnight. The following day, cells were washed with PBS, fixed with 4% (v/v) PFA for 5 min and washed again with PBS before permeabilization with 0.05% (v/v) Triton in PBS for 5 min. Following three washes, cells were incubated in 5% (v/v) donkey serum in PBS used as a blocking buffer. Coverslips were then incubated overnight at 4 °C with anti-iNOS (Abcam, ab49999; diluted at 1:500), anti-Hsp60 (Abcam, ab46798; diluted at 1:200), anti-mouse IgG (Cell Signaling Technology, 5415S; diluted at 1:1,000) or anti-rabbit IgG (Cell Signaling Technology, 3900S; diluted at 1:500) used as controls. The next day, coverslips were washed and incubated with donkey AF488 anti-mouse (ThermoFisher, 32766TR) or donkey AF680 anti-rabbit (ThermoFisher; A32788), both diluted at 1:500. After PBS washes, coverslips were attached to slides using 20 µl of Fluoromount-G mounting medium containing DAPI. Images of labelled fixed cells were captured using AiryScan microscopy under the ×60 oil objective at high resolution. Colocalization analyses were performed on the obtained images using the ZEN Blue software colocalization tool with the same threshold determined from the control condition (WT M1) and applied to each image and condition on the same *z*-stack.

### Structural modelling

Structural predictions of the (IRG1)_2_–(iNOS)_2_ heterotetramer were carried out using AlphaFold Multimer (v.2.3)^44^. Following the observation that predictions using the full UniProt sequences exhibited a large proportion of disordered regions (defined as long structured loops with predicted local distance difference test scores of <60) that exhibited notable variability across replicates, the terminal ends of both proteins were trimmed manually using PyMOL, leading to stable predictions. The constructs used in the UniProt numbering were IRG1_MOUSE 1-460 and NOS2_MOUSE 77-1,058, IRG1_HUMAN 1-460 and NOS2_HUMAN 84-1145. In the constructs involving calmodulin, the full sequence of CALM1_MOUSE or CALM1_HUMAN, respectively, was used. Predictions were run using the protocol described in the original publication^54^, including sequence information from UniRef as well as BFD and Mgnify, and templates available in the Protein Data Bank. All predictions were run on a NVIDIA A100 80 GB GPU. While this article was under review, an improved version of AlphaFold, AlphaFold 3 (ref. ^54^), was released to the public. Our predictions were subsequently compared with the more recent AlphaFold 3 server, and the results were found to be nearly identical.

### Molecular dynamics simulations

Molecular dynamics simulations were carried out starting from predicted AlphaFold Multimer (v.2.3) structures generated as described above, where iNOS is predicted to be in the input state^30^. *Cis*-peptide bonds and chiral centres with incorrect stereochemistry were identified and corrected using the Cispeptide and Chirality plugins of VMD^55^. Complexes were then solvated in orthorhombic boxes with a minimum of 1.4 nm between protein atoms and the box edge, and the protonation states were adjusted to pH 7.2 using the H++ server^56^. Counterions of Na^+^ and Cl^−^ were added to match an ionic force of 0.1 M using the SPLIT method^57^. All simulations were carried out using the AMBER ff94SB force field for protein atoms^58^, the TIP3P model for rigid water molecules^59^, the parameters for the haem group reported previously^60^, the parameters for flavin mononucleotide and flavin adenine dinucleotide reported previously^61^ and the General Amber Force Field (GAFF) for tetrahydrobiopterin ligands, with atomic charges derived from the minimized AM1 structures using ANTECHAMBER^62^. Pressure was fixed to 1 bar using a Monte Carlo barostat, and temperature was set using the Langevin thermostat. Before production simulations were carried out, complexes were minimized and then heated to 298 K at constant volume, with protein atoms restrained. These restraints were relaxed over a set of five 100 ps simulations at constant pressure, culminating with 100 ps of unrestrained equilibration. The production simulations used to produce the results shown in this paper used three simulation replicates of 300 ns that were run using OpenMM 7 on NVIDIA Quadro RTX 6000 GPUs^63^.

### Free energy calculations

Free energy calculations were carried out using the molecular mechanics with generalized born and surface area solvation (MM/GBSA) method^64^. Each calculation used 30 replicates of 5 ns equilibrium trajectories, started from a relaxed and equilibrated structure extracted from long molecular dynamics simulations. Each of the 30 replicates was subject to the same minimization, heating and equilibration procedure described in the section above. Free energies were calculated using the MMGBSA.py programme in AmberTools^65^, and 100 frames were collected every 40 ps from the final 4 ns of each 5 ns trajectory (that is, 3,000 frames per complex). Per-residue energy contributions to the binding energy were computed using the MMPBSA.py DECOMP functionality.

### Surface plasma resonance

The surface plasma resonance experiments were performed using a Biacore X100 equipped with a CM5 sensor (Cytiva, BR100012) chip. The CM5 sensor chips were immobilized with the IRG1 or iNOS using an amine-coupling kit (Cytiva, BR100050). The process was performed using both human and mouse proteins, resulting in four immobilized chips. The flow cell surfaces were activated for 7 min with 0.1 M NHS (*N*-hydroxysuccinimide) and 0.4 M EDC (1-ethyl-3-(3-dimethylaminopropyl)-carbodiimide 3) at a flow rate of 10 μl min^−1^. IRG1 and iNOS were diluted in 10 mM sodium acetate, pH 4.0 and pH 5.0, respectively. IRG1 and iNOS were immobilized onto different chips at a density of 3,423 RU and 200 RU, respectively, onto flow cell 2. Flow cell 1 was left blank to enable a blank correction of the sensograms. Both surfaces were blocked with a 7 min injection of 1 M ethanolamine-HCl, pH 8.5. All analyte proteins were dialysed into 1× HBS P+ buffer (Cytiva, BR100671) to match the running buffer used for analysis. A multi-cycle kinetics analysis was performed in duplicate for each ligand and respective analyte using twofold dilutions ranging from 25 nM to 200 nM. The association and dissociation time for each injection was 120 s and 600 s, respectively, followed by a regeneration using a 20 s injection of glycine 3 and a 30 s stabilization period. The blank-subtracted data were fit to a 1:1 interaction model using the Biacore Evaluation software. An eNOS chip was prepared and tested with IRG1 following the same protocol as the iNOS chip. Moreover, to confirm binding specificity, BSA (10 µg ml^−1^) was included as a negative control. Mouse iNOS was acquired from Cayman chemicals (60864), human iNOS from Origene (TP311819) and both human and mouse IRG1 were purified as previously described (see ‘Purification of Mouse Irg1’).

### Blue native gels

Cell lysates were prepared by homogenization in ice-cold non-denaturing cell lysis buffer (Cell Signaling Technology) containing fresh 1 mM PMSF or in CellLytic M buffer. Lysates were centrifuged at 17,000*g* for 10 min at 4 °C, and samples were prepared using Native PAGE buffer with 1% digitonin (Invitrogen). Then, 5 µg of protein was loaded by well in a 4–12% NuPAGE gel (Thermofisher) and run at 4 °C using Native PAGE running buffer and Native Blue for 30 min at 150 V, followed by 60 min at 200 V. Proteins were then transferred to a nitrocellulose membrane, which was then blocked with 5% (w/v) milk in PBS with 0.5% (v/v) Tween. Membranes were then exposed sequentially to anti-iNOS (BD Biosciences, 610431; diluted at 1:1,000), followed by anti-mouse IgG (H + L) and HRP conjugate (Promega, W4028; diluted at 1:25,000). Proteins were detected using the ChemiDoc Imaging system (Bio-Rad) following Amersham ECL Select western blotting detection reagent (Cytiva, RPN2235).

### Statistical analysis

All statistical analyses were carried out using Microsoft Excel (Microsoft) and GraphPad Prism 8 (GraphPad) software. Data are expressed as mean; error bars, s.e.m. or s.d. One-way ANOVA was used to compare multiple data groups affected by one single variable, with Dunnett’s test to compare each group with each other. Two-way ANOVA was used to compare multiple data groups affected by two independent variables, with Tukey’s post hoc test used to compare groups with each other. Statistical significance was indicated as ****P < 0.0001; ***P < 0.001; **P < 0.005; *P < 0.05; NS, not significant.

### Reporting summary

Further information on research design is available in the Nature Portfolio Reporting Summary linked to this article.

## Supplementary information

Reporting Summary

Supplementary Video 1 Molecular dynamics simulation of the murine heterotetramer (formed by the interaction between an iNOS dimer and an IRG1 dimer). This video represents one 300 ns simulation replicate.

Supplementary Video 2 Molecular dynamics simulation of the murine heterotetramer (formed by the interaction between an iNOS dimer and an IRG1 dimer). This video represents one 300 ns simulation replicate.

Supplementary Video 3 Molecular dynamics simulation of the murine heterotetramer (formed by the interaction between an iNOS dimer and an IRG1 dimer). This video represents one 300 ns simulation replicate.

Supplementary Video 4 Molecular dynamics simulation of the human heterotetramer (formed by the interaction between an iNOS dimer and an IRG1 dimer). This video represents one 300 ns simulation replicate.

Supplementary Video 5 Molecular dynamics simulation of the human heterotetramer (formed by the interaction between an iNOS dimer and an IRG1 dimer). This video represents one 300 ns simulation replicate.

Supplementary Video 6 Molecular dynamics simulation of the human heterotetramer (formed by the interaction between an iNOS dimer and an IRG1 dimer). This video represents one 300 ns simulation replicate.

## Source data

Source Data Fig. 1 Source data, unprocessed western blots.

Source Data Fig. 2 Source data.

Source Data Fig. 3 Source data, unprocessed western blots.

Source Data Fig. 4 Source data, unprocessed western blots.

Source Data Extended Data Fig. 1 Source data, unprocessed western blots.

Source Data Extended Data Fig. 2 Source data.

Source Data Extended Data Fig. 3 Source data, unprocessed western blots.

Source Data Extended Data Fig. 4 Source data, unprocessed western blots.

Source Data Extended Data Fig. 5 Source data, unprocessed western blots.

Source Data Extended Data Fig. 8 Source data, unprocessed western blots.

## Figures and Tables

**Extended Data Table 1:**
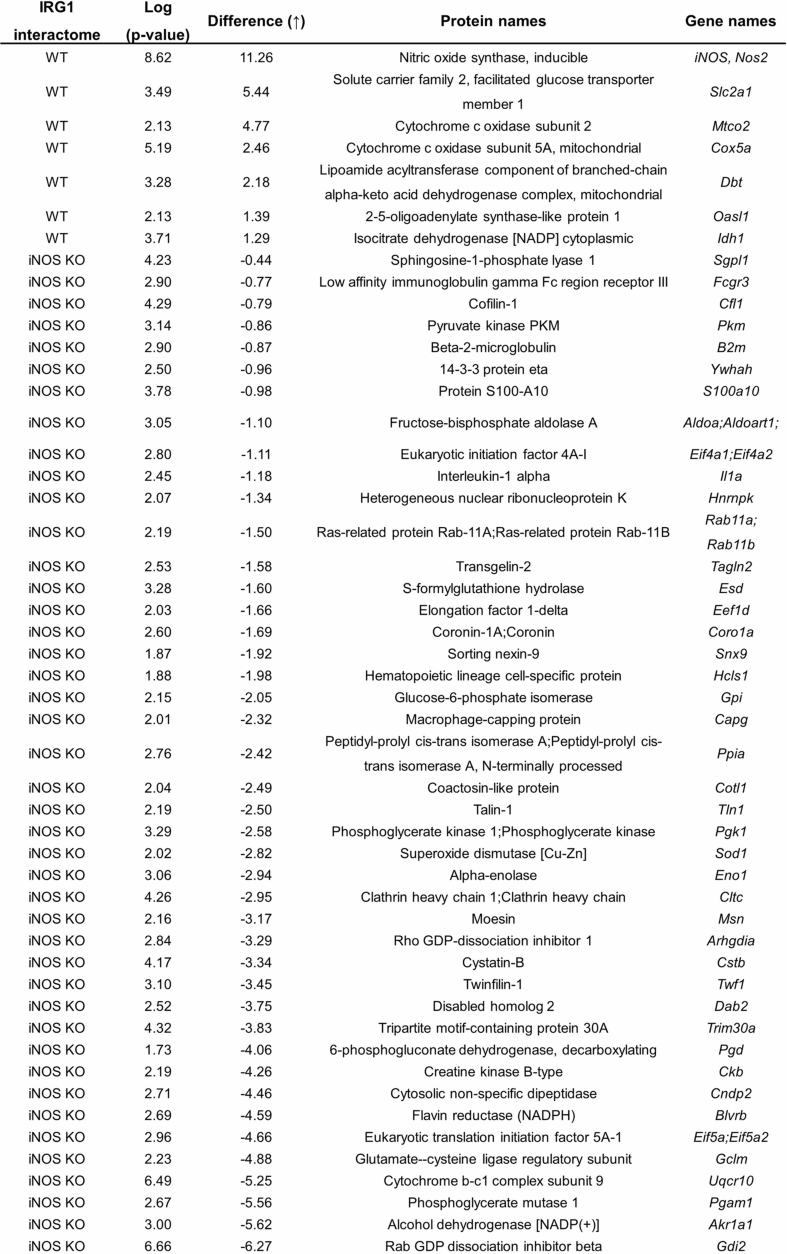
IRG1 interactome in LPS/IFNγ iNOS KO versus WT BMDMs

Only proteins interacting with IRG1 in WT or iNOS KO are shown (two tailed t-test cut off was set at 0.05).

**Extended Data Table 2:**
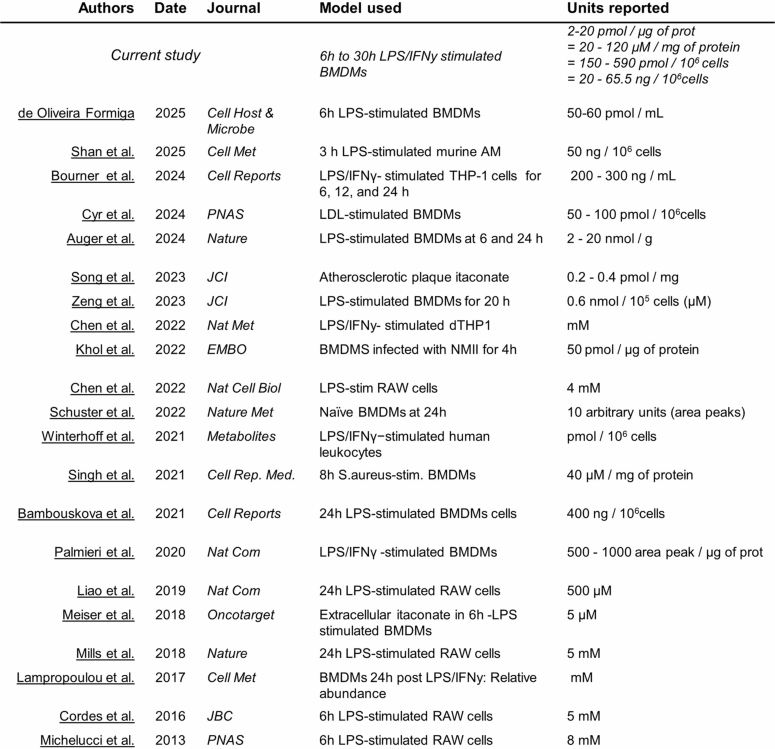
Itaconate units reported in the current literature

## Data Availability

All data are available in the main text or the supplementary materials. Proteomic data collected from the Co-IP of IRG1 by mass spectrometry have been uploaded to PRIDE (project accession no. PXD048712). Source data are provided with this paper.

## References

[1] Bailey, J. D. et al. Nitric oxide modulates metabolic remodeling in inflammatory macrophages through TCA cycle regulation and itaconate accumulation. *Cell Rep.* **28**, 218–230.e7 (2019). 31269442 10.1016/j.celrep.2019.06.018 PMC6616861

[2] Palmieri, E. M. et al. Nitric oxide orchestrates metabolic rewiring in M1 macrophages by targeting aconitase 2 and pyruvate dehydrogenase. *Nat. Commun.* **11**, 698 (2020). 32019928 10.1038/s41467-020-14433-7 PMC7000728

[3] Michelucci, A. et al. Immune-responsive gene 1 protein links metabolism to immunity by catalyzing itaconic acid production. *Proc. Natl Acad. Sci. USA* **110**, 7820–7825 (2013). 23610393 10.1073/pnas.1218599110 PMC3651434

[4] O’Neill, L. A. J. & Artyomov, M. N. Itaconate: the poster child of metabolic reprogramming in macrophage function. *Nat. Rev. Immunol.* **19**, 273–281 (2019). 30705422 10.1038/s41577-019-0128-5

[5] Cordes, T. et al. Immunoresponsive gene 1 and itaconate inhibit succinate dehydrogenase to modulate intracellular succinate levels. *J. Biol. Chem.* **291**, 14274–14284 (2016). 27189937 10.1074/jbc.M115.685792 PMC4933182

[6] Runtsch, M. C. et al. Itaconate and itaconate derivatives target JAK1 to suppress alternative activation of macrophages. *Cell Metab.* **34**, 487–501.e8 (2022). 35235776 10.1016/j.cmet.2022.02.002

[7] Bambouskova, M. et al. Electrophilic properties of itaconate and derivatives regulate the IκBζ–ATF3 inflammatory axis. *Nature* **556**, 501–504 (2018). 29670287 10.1038/s41586-018-0052-z PMC6037913

[8] Qin, W. et al. S-glycosylation-based cysteine profiling reveals regulation of glycolysis by itaconate. *Nat. Chem. Biol.* **15**, 983–991 (2019). 31332308 10.1038/s41589-019-0323-5

[9] Lin, J., Ren, J., Gao, D. S., Dai, Y. & Yu, L. The emerging application of itaconate: promising molecular targets and therapeutic opportunities. *Front. Chem.* **9**, 669308 (2021). 34055739 10.3389/fchem.2021.669308 PMC8149739

[10] McNeill, E. et al. Regulation of iNOS function and cellular redox state by macrophage *Gch1* reveals specific requirements for tetrahydrobiopterin in NRF2 activation. *Free Radic. Biol. Med.* **79**, 206–216 (2015). 25451639 10.1016/j.freeradbiomed.2014.10.575 PMC4344222

[11] Auger, J. P. et al. Metabolic rewiring promotes anti-inflammatory effects of glucocorticoids. *Nature* **629**, 184–192 (2024). 38600378 10.1038/s41586-024-07282-7

[12] Cordes, T. & Metallo, C. M. Itaconate alters succinate and coenzyme A metabolism via inhibition of mitochondrial complex II and methylmalonyl-CoA mutase. *Metabolites* **11**, 117 (2021). 33670656 10.3390/metabo11020117 PMC7922098

[13] Bailey, J. D. et al. Isolation and culture of murine bone marrow-derived macrophages for nitric oxide and redox biology. *Nitric Oxide* **100–101**, 17–29 (2020). 32339668 10.1016/j.niox.2020.04.005 PMC7284309

[14] Gross, T. J. et al. Epigenetic silencing of the human *NOS2* gene: rethinking the role of nitric oxide in human macrophage inflammatory responses. *J. Immunol.* **192**, 2326–2338 (2014). 24477906 10.4049/jimmunol.1301758 PMC3943971

[15] Chen, F., Kuhn, D. C., Gaydos, L. J. & Demers, L. M. Induction of nitric oxide and nitric oxide synthase mRNA by silica and lipopolysaccharide in PMA-primed THP-1 cells. *APMIS* **104**, 176–182 (1996). 8611191 10.1111/j.1699-0463.1996.tb00705.x

[16] Ozleyen, A., Yilmaz, Y. B. & Tumer, T. B. Dataset on the differentiation of THP-1 monocytes to LPS inducible adherent macrophages and their capacity for NO/iNOS signaling. *Data Brief* **35**, 106786 (2021). 33553532 10.1016/j.dib.2021.106786 PMC7851796

[17] Chang, Y. Y., Lu, C. W., Jean, W. H., Shieh, J. S. & Lin, T. Y. Phorbol myristate acetate induces differentiation of THP-1 cells in a nitric oxide-dependent manner. *Nitric Oxide* **109-110**, 33–41 (2021). 33667621 10.1016/j.niox.2021.02.002

[18] Weinberg, J. B. et al. Human mononuclear phagocyte inducible nitric oxide synthase (iNOS): analysis of iNOS mRNA, iNOS protein, biopterin, and nitric oxide production by blood monocytes and peritoneal macrophages. *Blood* **86**, 1184–1195 (1995). 7542498

[19] Kroncke, K. D., Fehsel, K. & Kolb-Bachofen, V. Inducible nitric oxide synthase in human diseases. *Clin. Exp. Immunol.* **113**, 147–156 (1998). 9717962 10.1046/j.1365-2249.1998.00648.x PMC1905037

[20] Cinelli, M. A., Do, H. T., Miley, G. P. & Silverman, R. B. Inducible nitric oxide synthase: regulation, structure, and inhibition. *Med. Res. Rev.* **40**, 158–189 (2020). 31192483 10.1002/med.21599 PMC6908786

[21] Khan, A. O. et al. Human bone marrow organoids for disease modeling, discovery, and validation of therapeutic targets in hematologic malignancies. *Cancer Discov.* **13**, 364–385 (2023). 36351055 10.1158/2159-8290.CD-22-0199 PMC9900323

[22] Poole, L. B. The basics of thiols and cysteines in redox biology and chemistry. *Free Radic. Biol. Med.* **80**, 148–157 (2015). 25433365 10.1016/j.freeradbiomed.2014.11.013 PMC4355186

[23] Bailey, J. et al. Tetrahydrobiopterin modulates ubiquitin conjugation to UBC13/UBE2N and proteasome activity by S-nitrosation. *Sci. Rep.* **8**, 14310 (2018). 30254268 10.1038/s41598-018-32481-4 PMC6156325

[24] Singh, S. P., Wishnok, J. S., Keshive, M., Deen, W. M. & Tannenbaum, S. R. The chemistry of the *S*-nitrosoglutathione/glutathione system. *Proc. Natl Acad. Sci. USA* **93**, 14428–14433 (1996). 8962068 10.1073/pnas.93.25.14428 PMC26149

[25] Swain, A. et al. Comparative evaluation of itaconate and its derivatives reveals divergent inflammasome and type I interferon regulation in macrophages. *Nat. Metab.* **2**, 594–602 (2020). 32694786 10.1038/s42255-020-0210-0 PMC7378276

[26] Degrandi, D., Hoffmann, R., Beuter-Gunia, C. & Pfeffer, K. The proinflammatory cytokine-induced IRG1 protein associates with mitochondria. *J. Interferon Cytokine Res.* **29**, 55–67 (2009). 19014335 10.1089/jir.2008.0013

[27] Webb, J. L., Harvey, M. W., Holden, D. W. & Evans, T. J. Macrophage nitric oxide synthase associates with cortical actin but is not recruited to phagosomes. *Infect. Immun.* **69**, 6391–6400 (2001). 11553583 10.1128/IAI.69.10.6391-6400.2001 PMC98774

[28] Chen, F. et al. Crystal structure of *cis*-aconitate decarboxylase reveals the impact of naturally occurring human mutations on itaconate synthesis. *Proc. Natl Acad. Sci. USA* **116**, 20644–20654 (2019). 31548418 10.1073/pnas.1908770116 PMC6789909

[29] Crane, B. R. et al. Structures of the *N*^ω^-hydroxy-l-arginine complex of inducible nitric oxide synthase oxygenase dimer with active and inactive pterins. *Biochemistry* **39**, 4608–4621 (2000). 10769116 10.1021/bi992409a

[30] Jiang, T., Zhang, H., Da Silva, G. M., Gyawali, Y. P. & Feng, C. Deciphering mutational effects on inducible NO synthase conformational dynamics via quantitative cross-linking mass spectrometry and AlphaFold2 subsampling. *J. Biol. Chem.* **301**, 110673 (2025). 40889679 10.1016/j.jbc.2025.110673 PMC12597282

[31] Akdel, M. et al. A structural biology community assessment of AlphaFold2 applications. *Nat. Struct. Mol. Biol.* **29**, 1056–1067 (2022). 36344848 10.1038/s41594-022-00849-w PMC9663297

[32] Sala, D., Engelberger, F., McHaourab, H. S. & Meiler, J. Modeling conformational states of proteins with AlphaFold. *Curr. Opin. Struct. Biol.* **81**, 102645 (2023). 37392556 10.1016/j.sbi.2023.102645

[33] Aoyagi, M. et al. Structures of tetrahydrobiopterin binding-site mutants of inducible nitric oxide synthase oxygenase dimer and implicated roles of Trp457. *Biochemistry* **40**, 12826–12832 (2001). 11669619 10.1021/bi011183k

[34] Gautier, C. et al. Dynamic regulation of the inducible nitric-oxide synthase by NO: comparison with the endothelial isoform. *J. Biol. Chem.* **279**, 4358–4365 (2004). 14594819 10.1074/jbc.M305048200

[35] Ghosh, S. et al. Mutational analysis of the tetrahydrobiopterin-binding site in inducible nitric-oxide synthase. *J. Biol. Chem.* **274**, 24100–24112 (1999). 10446182 10.1074/jbc.274.34.24100

[36] Bryk, R. & Wolff, D. J. Mechanism of inducible nitric oxide synthase inactivation by aminoguanidine and l-*N*^6^-(1-iminoethyl)lysine. *Biochemistry* **37**, 4844–4852 (1998). 9538001 10.1021/bi972065t

[37] Wolff, D. J. & Lubeskie, A. Aminoguanidine is an isoform-selective, mechanism-based inactivator of nitric oxide synthase. *Arch. Biochem. Biophys.* **316**, 290–301 (1995). 7530937 10.1006/abbi.1995.1040

[38] Cho, H. J., Martin, E., Xie, Q. W., Sassa, S. & Nathan, C. Inducible nitric oxide synthase: identification of amino acid residues essential for dimerization and binding of tetrahydrobiopterin. *Proc. Natl Acad. Sci. USA* **92**, 11514–11518 (1995). 8524794 10.1073/pnas.92.25.11514 PMC40432

[39] Zeng, Y. R. et al. The immunometabolite itaconate stimulates OXGR1 to promote mucociliary clearance during the pulmonary innate immune response. *J. Clin. Invest.* **133**, e160463 (2023). 36919698 10.1172/JCI160463 PMC10014103

[40] Su, C., Cheng, T., Huang, J., Zhang, T. & Yin, H. 4-Octyl itaconate restricts STING activation by blocking its palmitoylation. *Cell Rep.* **42**, 113040 (2023). 37624697 10.1016/j.celrep.2023.113040

[41] Skwarczynska, M. & Ottmann, C. Protein–protein interactions as drug targets. *Future Med. Chem.* **7**, 2195–2219 (2015). 26510391 10.4155/fmc.15.138

[42] Gu, Y. & Zhu, D. nNOS-mediated protein–protein interactions: promising targets for treating neurological and neuropsychiatric disorders. *J. Biomed. Res.* **35**, 1–10 (2020). 33402546 10.7555/JBR.34.20200108 PMC7874267

[43] Su, Y. Regulation of endothelial nitric oxide synthase activity by protein–protein interaction. *Curr. Pharm. Des.* **20**, 3514–3520 (2014). 24180383 10.2174/13816128113196660752 PMC7039309

[44] Li, X., Liu, W., Huang, X., Xiong, J. & Wei, X. Interaction of AR and iNOS in lens epithelial cell: a new pathogenesis and potential therapeutic targets of diabetic cataract. *Arch. Biochem. Biophys.* **615**, 44–52 (2017). 28108234 10.1016/j.abb.2017.01.007

[45] Chuaiphichai, S. et al. Cell-autonomous role of endothelial GTP cyclohydrolase 1 and tetrahydrobiopterin in blood pressure regulation. *Hypertension* **64**, 530–540 (2014). 24777984 10.1161/HYPERTENSIONAHA.114.03089 PMC5238938

[46] Diotallevi, M. et al. Isolation and in vitro culture of bone marrow-derived macrophages for the study of NO-redox biology. *J. Vis. Exp.*10.3791/62834 (2022). 10.3791/62834 35723458

[47] Olijnik, A. A. et al. Generating human bone marrow organoids for disease modeling and drug discovery. *Nat. Protoc.* **19**, 2117–2146 (2024). 38532070 10.1038/s41596-024-00971-7

[48] Yuhara, K., Yonehara, H., Hattori, T., Kobayashi, K. & Kirimura, K. Enzymatic characterization and gene identification of aconitate isomerase, an enzyme involved in assimilation of trans-aconitic acid, from *Pseudomonas* sp. WU-0701. *FEBS J.* **282**, 4257–4267 (2015). 26293748 10.1111/febs.13494

[49] Huang, X., Lu, X., Li, Y., Li, X. & Li, J. J. Improving itaconic acid production through genetic engineering of an industrial *Aspergillus terreus* strain. *Microb. Cell Fact.* **13**, 119 (2014). 25162789 10.1186/s12934-014-0119-y PMC4251695

[50] Dwiarti, L., Yamane, K., Yamatani, H., Kahar, P. & Okabe, M. Purification and characterization of *cis*-aconitic acid decarboxylase from *Aspergillus terreus* TN484-M1. *J. Biosci. Bioeng.* **94**, 29–33 (2002). 16233265 10.1263/jbb.94.29

[51] Vuoristo, K. S. et al. Heterologous expression of *Mus musculus* immunoresponsive gene 1 (*irg1*) in *Escherichia coli* results in itaconate production. *Front. Microbiol.* **6**, 849 (2015). 26347730 10.3389/fmicb.2015.00849 PMC4539527

[52] Walsby-Tickle, J. et al. Anion-exchange chromatography mass spectrometry provides extensive coverage of primary metabolic pathways revealing altered metabolism in IDH1 mutant cells. *Commun. Biol.* **3**, 247 (2020). 32433536 10.1038/s42003-020-0957-6 PMC7239943

[53] Krasnoselska, G. O. et al. Transient transfection and expression of eukaryotic membrane proteins in Expi293F cells and their screening on a small scale: application for structural studies. *Methods Mol. Biol.* **2305**, 105–128 (2021). 33950386 10.1007/978-1-0716-1406-8_5

[54] Jumper, J. et al. Highly accurate protein structure prediction with AlphaFold. *Nature* **596**, 583–589 (2021). 34265844 10.1038/s41586-021-03819-2 PMC8371605

[55] Schreiner, E., Trabuco, L. G., Freddolino, P. L. & Schulten, K. Stereochemical errors and their implications for molecular dynamics simulations. *BMC Bioinformatics* **12**, 190 (2011). 21605430 10.1186/1471-2105-12-190 PMC3124434

[56] Gordon, J. C. et al. H++: a server for estimating p*K*_a_s and adding missing hydrogens to macromolecules. *Nucleic Acids Res.* **33**, W368–W371 (2005). 15980491 10.1093/nar/gki464 PMC1160225

[57] Machado, M. R. & Pantano, S. Split the charge difference in two! A rule of thumb for adding proper amounts of ions in MD simulations. *J. Chem. Theory Comput.* **16**, 1367–1372 (2020). 31999456 10.1021/acs.jctc.9b00953

[58] Maier, J. A. et al. ff14SB: Improving the accuracy of protein side chain and backbone parameters from ff99SB. *J. Chem. Theory Comput.* **11**, 3696–3713 (2015). 26574453 10.1021/acs.jctc.5b00255 PMC4821407

[59] Jorgensen, W. L., Chandrasekhar, J., Madura, J. D., Impey, R. W. & Klein, M. L. Comparison of simple potential functions for simulating liquid water. *J. Chem. Phys.* **79**, 926–935 (1983).

[60] Shahrokh, K., Orendt, A., Yost, G. S. & Cheatham, T. E. III Quantum mechanically derived AMBER-compatible heme parameters for various states of the cytochrome P450 catalytic cycle. *J. Comput. Chem.* **33**, 119–133 (2012). 21997754 10.1002/jcc.21922 PMC3242737

[61] Dupradeau, F. Y. et al. R.E.DD.B.: a database for RESP and ESP atomic charges, and force field libraries. *Nucleic Acids Res.* **36**, D360–D367 (2008). 17962302 10.1093/nar/gkm887 PMC2238896

[62] Wang, J., Wang, W., Kollman, P. A. & Case, D. A. Automatic atom type and bond type perception in molecular mechanical calculations. *J. Mol. Graph. Model.* **25**, 247–260 (2006). 16458552 10.1016/j.jmgm.2005.12.005

[63] Eastman, P. et al. OpenMM 7: rapid development of high performance algorithms for molecular dynamics. *PLoS Comput. Biol.* **13**, e1005659 (2017). 28746339 10.1371/journal.pcbi.1005659 PMC5549999

[64] Ylilauri, M. & Pentikainen, O. T. MMGBSA as a tool to understand the binding affinities of filamin–peptide interactions. *J. Chem. Inf. Model.* **53**, 2626–2633 (2013). 23988151 10.1021/ci4002475

[65] Miller, B. R. et al. MMPBSA.py: an efficient program for end-state free energy calculations. *J. Chem. Theory Comput.* **8**, 3314–3321 (2012). 26605738 10.1021/ct300418h

