# Supplementary material for: iNOS modulates inflammatory responses in an NO-independent manner through direct interaction with IRG1 in mitochondria
Source: Nat Metab. 2026 Apr 10;8(4):855–68. doi: 10.1038/s42255-026-01492-1 (PMC13121041; doi:10.1038/s42255-026-01492-1)
Supplement: Supplementary file 1 — Reporting Summary [file 42255_2026_1492_MOESM1_ESM.pdf]

nature portfolio

Corresponding author(s): Marina Diotallevi
Mark Crabtree
Keith Channon

Last updated by author(s): 20/11/2025

Reporting Summary

Nature Portfolio wishes to improve the reproducibility of the work that we publish. This form provides structure for consistency and transparency in reporting. For further information on Nature Portfolio policies, see our Editorial Policies and the Editorial Policy Checklist.

Statistics

For all statistical analyses, confirm that the following items are present in the figure legend, table legend, main text, or Methods section.

n/a	Confirmed	
☐	☒	The exact sample size (n) for each experimental group/condition, given as a discrete number and unit of measurement
☐	☒	A statement on whether measurements were taken from distinct samples or whether the same sample was measured repeatedly
☐	☒	The statistical test(s) used AND whether they are one- or two-sided<br>*Only common tests should be described solely by name; describe more complex techniques in the Methods section.*
☒	☐	A description of all covariates tested
☐	☒	A description of any assumptions or corrections, such as tests of normality and adjustment for multiple comparisons
☐	☒	A full description of the statistical parameters including central tendency (e.g. means) or other basic estimates (e.g. regression coefficient) AND variation (e.g. standard deviation) or associated estimates of uncertainty (e.g. confidence intervals)
☐	☒	For null hypothesis testing, the test statistic (e.g. F, t, r) with confidence intervals, effect sizes, degrees of freedom and P value noted<br>*Give P values as exact values whenever suitable.*
☒	☐	For Bayesian analysis, information on the choice of priors and Markov chain Monte Carlo settings
☒	☐	For hierarchical and complex designs, identification of the appropriate level for tests and full reporting of outcomes
☐	☒	Estimates of effect sizes (e.g. Cohen's d, Pearson's r), indicating how they were calculated

Our web collection on statistics for biologists contains articles on many of the points above.

Software and code

Policy information about availability of computer code

Data collection	Software used to collect data:<br>• HPLC: ChromNAV 2.0 HPLC software by Jasco<br>• RT-qPCR: QuantStudioTM Real-Time PCR Software v1.7.2 by Thermo Fisher Scientific<br>• Western blot: LI-COR Image Studio by LICOR<br>• Griess assay, BCA protein assay, CellTox Green cytotoxicity assay: CLARIOstar by BMG Labtech<br>• Hydrogen peroxide measurements: LabScribe Software for LabTrax 2 by World Precision Instruments<br>• Mass spectrometry: MaxQuant (Version 1.6.17.0) / Perseus for data analysis<br>• Airyscan imaging: ZEN Blue software by ZEISS<br>• Surface plasma resonance: Biacore X100 Evaluation, Version 2.0.2 by Cytiva<br>• Structural modelling: AlphaFold Multimer v2.3<br>• Molecular dynamics simulations: OpenMM 7 on NVIDIA Quadro RTX 6000 GPUs<br>• Free energy calculations: MMGBSA.py program in AmberTools
Data analysis	All statistical analyses were carried out using Microsoft Excel (Microsoft) and GraphPad Prism (GraphPad Inc., Version 10.6.1) software.

For manuscripts utilizing custom algorithms or software that are central to the research but not yet described in published literature, software must be made available to editors and reviewers. We strongly encourage code deposition in a community repository (e.g. GitHub). See the Nature Portfolio guidelines for submitting code & software for further information.

Data

Policy information about availability of data

All manuscripts must include a data availability statement. This statement should provide the following information, where applicable:

- Accession codes, unique identifiers, or web links for publicly available datasets
- A description of any restrictions on data availability
- For clinical datasets or third party data, please ensure that the statement adheres to our policy

Proteomic data collected from the co-immunoprecipitation of IRG1 by mass spectrometry has been uploaded to PRIDE (Project accession: PXD048712). Molecular dynamics simulations of the IRG1-iNOS complex can be found here: DOI 10.5281/zenodo.18643560. Data supporting other findings in this study are all available in the Source Data File.

Research involving human participants, their data, or biological material

Policy information about studies with human participants or human data. See also policy information about sex, gender (identity/presentation), and sexual orientation and race, ethnicity and racism.

Reporting on sex and gender	N/A: anonymous
Reporting on race, ethnicity, or other socially relevant groupings	N/A: anonymous
Population characteristics	N/A
Recruitment	Human blood was obtained from healthy donors in the form of leukocyte cones.
Ethics oversight	Informed consent and ethical approvals were obtained from the NHS Blood and Transplant service (UK).

Note that full information on the approval of the study protocol must also be provided in the manuscript.

Field-specific reporting

Please select the one below that is the best fit for your research. If you are not sure, read the appropriate sections before making your selection.

☒ Life sciences ☐ Behavioural & social sciences ☐ Ecological, evolutionary & environmental sciences

For a reference copy of the document with all sections, see nature.com/documents/nr-reporting-summary-flat.pdf

Life sciences study design

All studies must disclose on these points even when the disclosure is negative.

Sample size	No statistical methods were used to predetermine sample size due to the exploratory nature of the study. The selected sample sizes are in line with standard practice in the field and were sufficient to produce consistent and statistically meaningful results across independent experiments. For molecular assays, a minimum of three independent experiments were conducted. For experiments involving primary cells (mice or human donors), a minimum of four biological replicates were used.
Data exclusions	No data was excluded unless it fails due to technical reason.
Replication	Each experiment was replicated at least three times. Each replication was successful.
Randomization	Samples were allocated to experimental groups based on genotype and/or experimental condition. Randomization was not applicable because group assignment was inherently determined by genotype or treatment. Covariates related to animals were controlled by matching animals where possible and by processing all samples in parallel.
Blinding	Investigators were not blinded in this study. For experiments involving Western blotting and transfections, all genotypes / experimental conditions were processed in parallel to ensure equal representation. For other molecular assays, sample coding during data collection and automated or predefined analysis pipelines were used where possible to ensure objective data analysis.

Reporting for specific materials, systems and methods

We require information from authors about some types of materials, experimental systems and methods used in many studies. Here, indicate whether each material, system or method listed is relevant to your study. If you are not sure if a list item applies to your research, read the appropriate section before selecting a response.

Materials & experimental systems

n/a	Involved in the study
☐	☒ Antibodies
☐	☒ Eukaryotic cell lines
☒	☐ Palaeontology and archaeology
☐	☒ Animals and other organisms
☒	☐ Clinical data
☒	☐ Dual use research of concern
☒	☐ Plants

Methods

n/a	Involved in the study
☒	☐ ChIP-seq
☒	☐ Flow cytometry
☒	☐ MRI-based neuroimaging

Antibodies

Antibodies used

Anti-iNOS (BD Biosciences; 610431; diluted at 1:1000 for WB)
Anti-iNOS (Abcam; ab49999; diluted at 1:1000 for WB and 1:500 for IF)
Anti-IRG1 (Abcam; ab222411; diluted at 1:1000 for WB)
Anti-GAPDH (Merck; MAB374; diluted at 1:1000 for WB)
Anti-VDAC (Cell Signaling Technology; 4661S; diluted at 1:1000 for WB)
Anti-Hsp60 (Abcam; ab46798, diluted at 1:200 for IF)
Rabbit (DA1E) mAb IgG XP® Isotype Control #3900; Cell Signaling Technology; diluted at 1:500 for IF
Mouse (G3A1) mAb IgG1 Isotype Control #5415; Cell Signaling Technology; diluted at 1:1000 for IF
Donkey anti-Mouse IgG (H+L) Highly Cross-Adsorbed Secondary Antibody, Alexa Fluor™ Plus 488 diluted at 1:500 for IF (ThermoFisher; A32766TR)
Donkey anti-Mouse IgG (H+L) Highly Cross-Adsorbed Secondary Antibody, Alexa Fluor™ Plus 680 diluted at 1:500 for IF (ThermoFisher; A32788)
IRDye680RD Goat anti-Mouse (LICORbio; 926-68070; diluted at 1:25,000)
IRDye488RD Goat anti-Rabbit (LICORbio; 926-32211; diluted at 1:25,000)
Anti-Mouse IgG (H+L), HRP Conjugate (Promega; W4028, diluted at 1:25,000)

Validation

All antibodies are commercially available and validated.

Eukaryotic cell lines

Policy information about cell lines and Sex and Gender in Research

Cell line source(s)

HEK-293T (HEKs)

Authentication

Cells were obtained from European Collection of Authenticated Cell Cultures (ECACC; Merck; Catalog No: 12022001).
Authenticated by STR-PCR Data:
Amelogenin: X
CSF1PO: 11,12
D13S317: 12
D16S539: 9,13
D5S818: 8,9
D7S820: 11
THO1: 7,9.3
TPOX: 11
vWA: 16,19

Mycoplasma contamination

Tested negative for mycoplasma contamination

Commonly misidentified lines
(See ICLAC register)

N/A

Animals and other research organisms

Policy information about studies involving animals; ARRIVE guidelines recommended for reporting animal research, and Sex and Gender in Research

Laboratory animals

Nos2−/− (Nos2tm1Lau) (iNOS KO) and wild-type C57BL6/J mice were purchased from The Jackson Laboratory. Experiments were performed using bone marrow isolated from 10-16 weeks old adult male and female.
Gch1fl/fl animals were bred with Tie2cre transgenic mice to produce Gch1fl/flTie2cre mice where Gch1 is deleted in endothelial cells and bone marrow-derived cells. The Tie2cre transgene is active in the female germline. Consequently, only male animals are used to establish breeding pairs to maintain conditional expression.
Experiments were performed using bone marrow isolated from 10-16 weeks old adult male and female Gch1fl/flTie2cre (referred to

	as Gchfl/flTie2cre) and their Gch1fl/fl (Gchfl/fl) littermates on a pure (> 10 generations) C57BL6/J background. For more details, please see: McNeill et al. (2015) and Chuaiphichai et al. (2014).
Wild animals	N/A
Reporting on sex	Experiments include both male and female mice.
Field-collected samples	Mice were housed in ventilated cages with a 12-h light/dark cycle and controlled temperature (20–22 °C), and fed normal chow and water ad libitum.
Ethics oversight	All animal procedures were approved and carried out in accordance with the University of Oxford ethical committee and the UK Home Office Animals (Scientific Procedures) Act 1986. All procedures conformed with the Directive 2010/63/EU of the European Parliament.

Note that full information on the approval of the study protocol must also be provided in the manuscript.

Plants

Seed stocks	*Report on the source of all seed stocks or other plant material used. If applicable, state the seed stock centre and catalogue number. If plant specimens were collected from the field, describe the collection location, date and sampling procedures.*
Novel plant genotypes	*Describe the methods by which all novel plant genotypes were produced. This includes those generated by transgenic approaches, gene editing, chemical/radiation-based mutagenesis and hybridization. For transgenic lines, describe the transformation method, the number of independent lines analyzed and the generation upon which experiments were performed. For gene-edited lines, describe the editor used, the endogenous sequence targeted for editing, the targeting guide RNA sequence (if applicable) and how the editor was applied.*
Authentication	*Describe any authentication procedures for each seed stock used or novel genotype generated. Describe any experiments used to assess the effect of a mutation and, where applicable, how potential secondary effects (e.g. second site T-DNA insertions, mosiacism, off-target gene editing) were examined.*
